# The iron chelator, PBT434, modulates transcellular iron trafficking in brain microvascular endothelial cells

**DOI:** 10.1371/journal.pone.0254794

**Published:** 2021-07-26

**Authors:** Danielle K. Bailey, Whitney Clark, Daniel J. Kosman

**Affiliations:** Department of Biochemistry, Jacobs School of Medicine and Biomedical Sciences, State University of New York at Buffalo, Buffalo, NY, United States of America; Lady Davis Institute for Medical Research, CANADA

## Abstract

Iron and other transition metals, such as copper and manganese, are essential for supporting brain function, yet over-accumulation is cytotoxic. This over-accumulation of metals, particularly iron, is common to several neurological disorders; these include Alzheimer’s disease, Parkinson’s disease, Friedrich’s ataxia and other disorders presenting with neurodegeneration and associated brain iron accumulation. The management of iron flux by the blood-brain barrier provides the first line of defense against the over-accumulation of iron in normal physiology and in these pathological conditions. In this study, we determined that the iron chelator PBT434, which is currently being developed for treatment of Parkinson’s disease and multiple system atrophy, modulates the uptake of iron by human brain microvascular endothelial cells (hBMVEC) by chelation of extracellular Fe^2+^. Treatment of hBMVEC with PBT434 results in an increase in the abundance of the transcripts for transferrin receptor (TfR) and ceruloplasmin (Cp). Western blot and ELISA analyses reveal a corresponding increase in the proteins as well. Within the cell, PBT434 increases the detectable level of chelatable, labile Fe^2+^; data indicate that this Fe^2+^ is released from ferritin. In addition, PBT434 potentiates iron efflux likely due to the increase in cytosolic ferrous iron, the substrate for the iron exporter, ferroportin. PBT434 equilibrates rapidly and bi-directionally across an hBMVEC blood-brain barrier. These results indicate that the PBT434-iron complex is not substrate for hBMVEC uptake and thus support a model in which PBT434 would chelate interstitial iron and inhibit re-uptake of iron by endothelial cells of the blood-brain barrier, as well as inhibit its uptake by the other cells of the neurovascular unit. Overall, this presents a novel and promising mechanism for therapeutic iron chelation.

## Introduction

Metal chelation therapy (MCT) has long been used as a treatment for transition metal poisoning and for genetic disorders in the metabolism of an essential metal ion that lead to the metal’s over-accumulation [1–3]. Two examples of the latter are the hyper-accumulation of copper in Wilson’s disease [4] and of iron in hereditary hemochromatosis [5]. Both copper and iron are catalysts of oxidative stress and thus are cytotoxic at concentrations that exceed the ability of the cell and organism to ‘chaperone’ these redox active transition metals [6, 7]. Iron accumulation, in particular, is broadly idiopathic; indeed, an increase in iron is a hallmark of an aging brain [8–10]. Pathologically, this brain iron accumulation is a feature of mutations in genes unrelated to iron metabolism [11–15] as well as a variety of other neurodegenerative diseases, some of which lack a specific genetic link such as aging [16], Alzheimer’s Disease [17], Friedreich’s Ataxia [18] and Parkinson’s Disease [19]. As a group, such disorders can be thought of as neurodegeneration with brain iron accumulation (NBIA) although this acronym commonly has been restricted to those for which a genetic link has been identified [11, 13, 14].

In the case of iron *over-load*, the objective is to ‘cleanse’ the body of excess iron due to a defect in cell iron uptake or efflux. Here the objective is to out-compete physiologic iron chelators with the drug; a compound that has good pharmacokinetics *and* high affinity for ferrous iron is the target drug. Since the body is over-replete with the essential metal, there is little concern about inducing a deficiency in the course of treatment. Treating cerebral disease by iron chelation therapy requires a different strategy. This is not a problem of systemic iron overload, but of iron accumulation in areas of pathology with damaging downstream sequelae. Age-associated iron accumulation in Parkinson’s disease (PD), for instance, potentially contributes to oxidative stress-related cellular damage [20]. Excessive labile iron promotes the misfolding of α-synuclein in substantia nigral neurons. The use of a high affinity chelator may lead to some reduction in the brain iron load but will most certainly induce an iron deficiency that in the aged population, at least, is contraindicated given the systemic iron deficiency common to that age group [21]. A chelator with an optimal affinity has the potential to reduce iron accumulation as well as the attendant oxidative stress due to excess labile iron and underlying disease processes.

One chelator approved for use in treating transfusion-induced iron over-load in thalassemia patients is deferiprone (DFP, brand name Ferriprox) [5, 22]. DFP also has been used in treating Friedreich’s ataxia [23] and Parkinson’s disease [24, 25]. In a meta-analysis, DFP has been shown to provide significant reductions in myocardial iron content as well as greater cardiac protection in thalassemia patients than deferoxamine, the classical iron chelating agent [5]. On the other hand, DFP is rapidly metabolized by the liver [26] and more recent work has shown it chelates Fe^2+^ at the active site of iron-dependent histone lysine demethylases, an activity that correlates with a previously unrecognized cytotoxicity [27]. This finding underscores a key limitation in the use of iron chelation therapy, namely competition by the drug for physiologically-essential iron, whether in an iron store or a protein that harbors a prosthetic iron species. None-the-less, DFP, for example, has shown efficacy in a Phase 2 trial treatment of Parkinson’s disease as indicated by both analytic (reduced brain iron load by T2*-weighted MRI) and behavioral indices (cognitive and motor neuron function) [24, 25].

The affinity of DFP for Fe^3+^ remains a concern, however. The stable DFP-iron species is the tris-complex, [Fe(DFP)_3_]^0^ [28]. While the neutrality of this complex is ideal for mobilizing iron out of the cell, the stability constant for it, ~10^37^, makes DFP a true iron scavenger; in this context its inhibition of an iron enzyme like lysine demethylase is predictable [27]. This concern reflects the need to develop iron chelators that have the membrane-permeability of DFP but a significantly *weaker* affinity for *both* Fe^2+^ and Fe^3+^. This latter feature limits drug scavenging of prosthetic metal *and* the thermodynamic potential of the chelating agent to catalyze the ferrous iron auto-oxidation that results in the production of reactive oxygen species. In essence, strong ferric iron chelators catalyze the pro-oxidant property of Fe^2+^ [29]. In this study we report how such an iron chelator with moderate ferric and ferrous iron affinities modulates the flux of iron in the brain microvascular endothelial cells that form the blood brain barrier (BBB).

**Fig 1:**
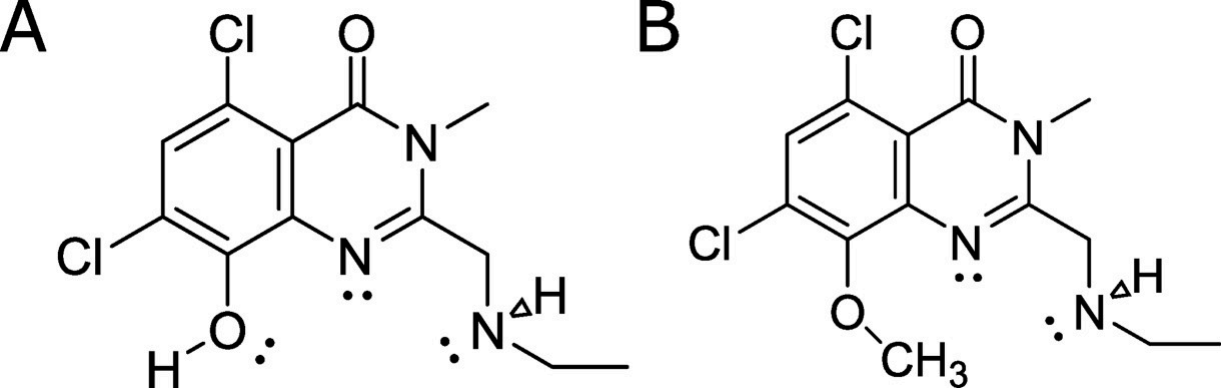
PBT434 and O-Methyl PBT434. (**A**) PBT434, 5,7-dichloro-2-((ethylamino)methyl)-8-hydroxy-3-methylquinazolin-4(3H)-one. (**B**) PBT434-met, the 8-methoxy, non-chelating derivative of PBT434. PBT434 forms a bis-complex with both Fe^2+^ and Fe^3+^.

This drug, PBT434 [5,7-dichloro-2-((ethylamino)methyl)-8-hydroxy-3-methylquinazolin-4(3H)-one, **Fig 1A**], forms a bis-iron complex with log stability constants of ~11 and ~15 for Fe^2+^ and Fe^3+^, respectively [30]. PBT434 prevented loss of substantia nigra pars compacta (SNpc) neurons, lowered nigral α-synuclein accumulation, reduced PD disease model-related iron content in the midbrain, and rescued motor performance in two mouse models of Parkinson’s disease without any apparent depletion of systemic iron stores [30]. PBT434 is also efficacious in murine models of Multiple System Atrophy (MSA) [30, 31], a motor disorder similar in presentation to Parkinson’s but which is characterized by α-synuclein misfolding and subsequent accumulation causing the formation of glial cytoplasmic inclusions that are the hallmark pathology of the disease [32]. Significantly, PBT434 reduced markers of oxidative stress in mouse PD models [30] indicating that 1) PBT434 targeted iron stores that otherwise were primed to function as pro-oxidants and 2) PBT434 did *not* potentiate this nascent oxidation-based cytotoxicity. PBT434 has completed a Phase 1 study satisfactorily [33].

The work presented here was designed to interrogate the impact PBT434 has on iron trafficking in the brain’s barrier cells, the microvascular endothelial cells that together with underlying glia form the blood brain barrier. These studies have used a well-validated immortalized endothelial cell line in both monolayer and transwell culture formats [34–37]. The primary objective of these studies was to determine the kinetics of iron uptake and efflux from these cells and their modulation by PBT434. The transwell BBB model was used also to demonstrate the *bi-directional* PBT434 transcellular flux across the endothelial cell barrier. The model demonstrated in molecular terms that PBT434 inhibits iron uptake by chelation, while stimulating iron efflux. Cell imaging studies indicate that PBT434 accesses the same labile iron pool probed by a classic Fe^2+^ chelating agent, 2,2’-bipyridine or bipyridyl, and a fluorescent probe for ferrous iron. The results suggest a possible mechanism of action for PBT434 that includes inhibition of uptake of systemic iron at the BBB, and subsequent sequestration of brain iron in the interstitial space.

## Results

### PBT434 has no cytotoxic effects on brain microvascular endothelial cells

**Fig 2:**
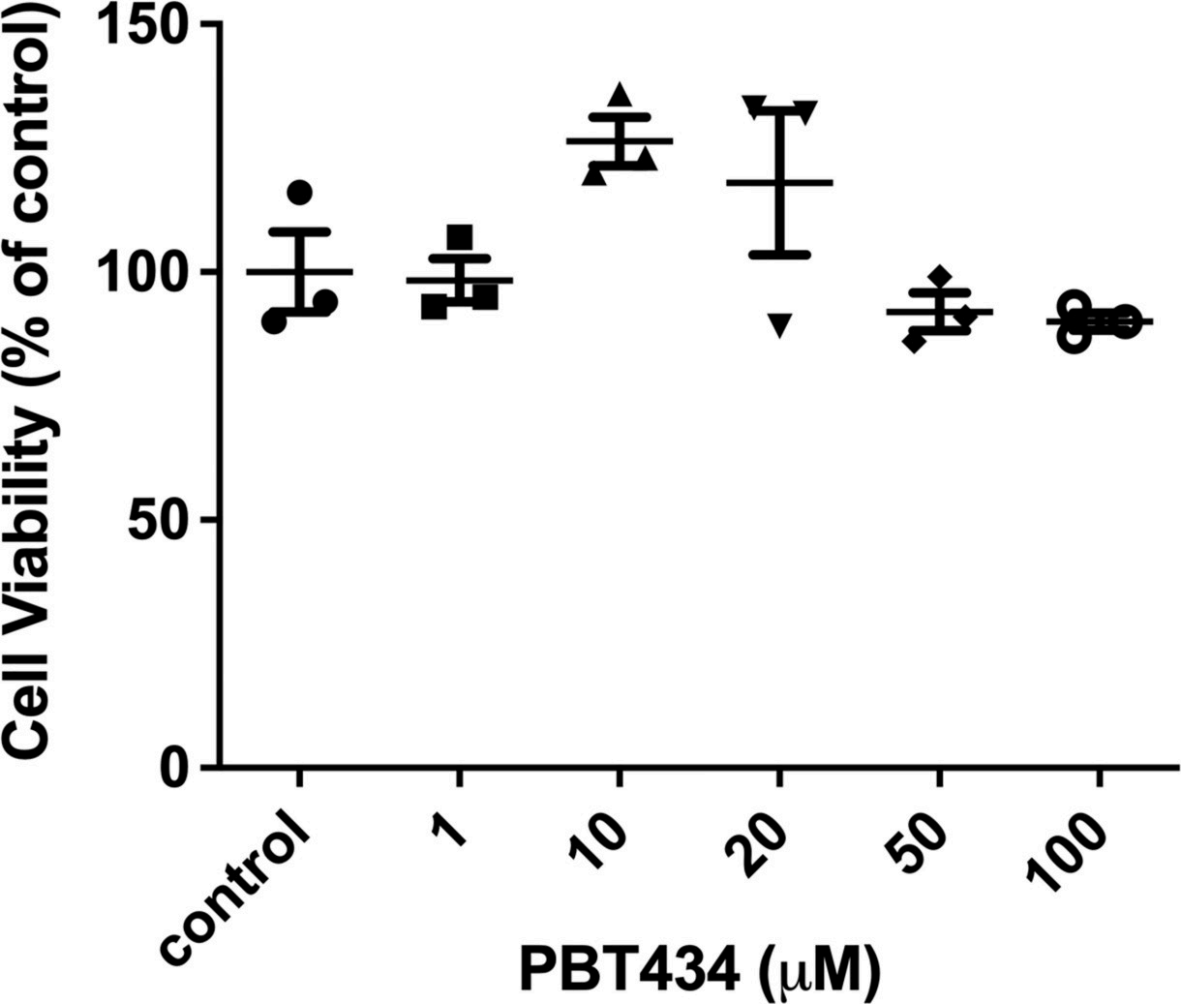
PBT434 toxicity in brain microvascular endothelial cells. hBMVEC were incubated with increasing concentrations of PBT434 for 24h. Cell viability was then assessed by standard MTT assay that interrogates physiologic mitochondrial function, using Triton-X as a positive control for cell killing. Data are represented as mean ± SEM, n = 3 biological replicates. Statistical significance was determined using Welch’s ANOVA with Dunnett’s T3 multiple comparison test compared to control.

To determine an appropriate range of working concentrations for PBT434 in our *in vitro* cell culture, we utilized the MTT assay to monitor hBMVEC mitochondrial function in response to PBT434. Based on previous reports [30], hBMVEC were treated with a range of PBT434 concentrations up to 100 μM for 24h. We observed no significant changes in hBMVEC viability with any concentration tested (**Fig 2**).

### PBT434 is rapidly taken up and trafficked across the hBMVEC barrier

**Fig 3:**
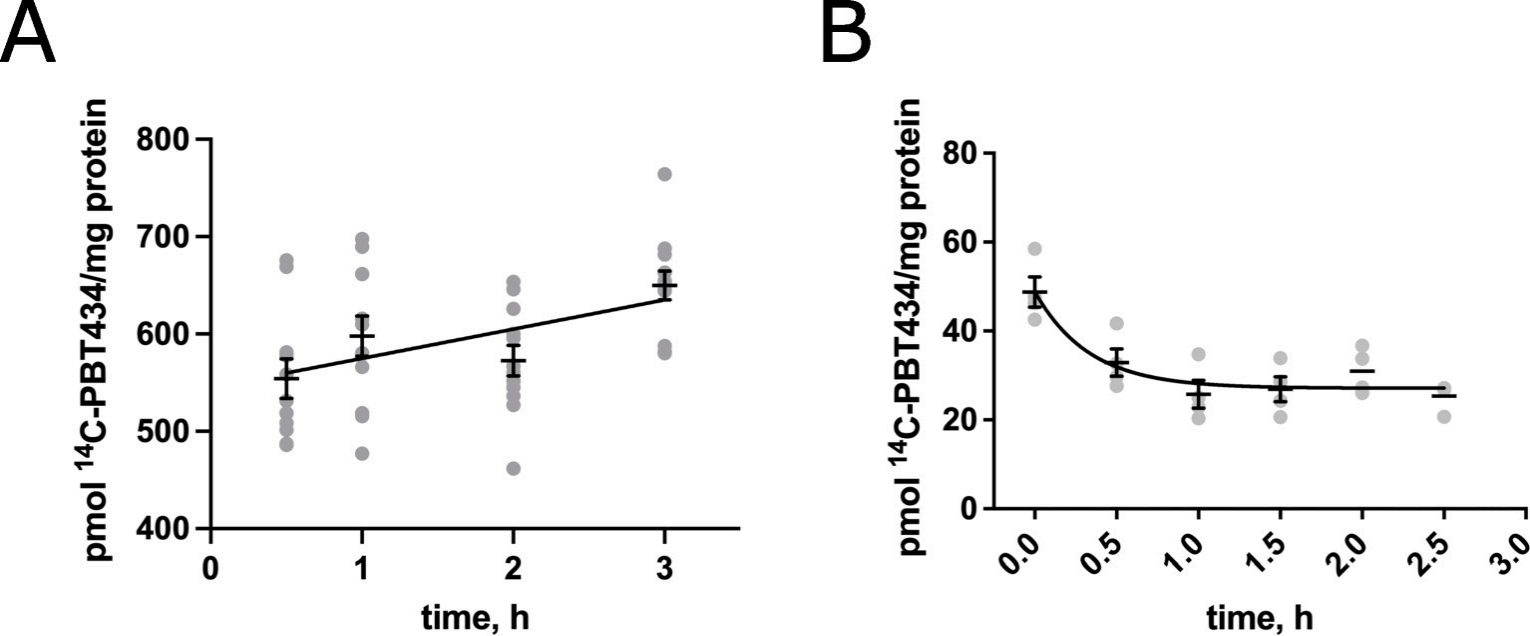
^14^C-PBT434 accumulation and efflux from hBMVEC. hBMVEC were loaded with 20 μM ^14^C-PBT434 at 37°C for 0.5-3h (**A**) and the amount of ^14^C-PBT434 accumulated in the lysates at each time point was plotted, n = 12 experimental replicates/time point. The rate of ^14^C-PBT434 accumulation from 0.5-3h was calculated using linear regression analysis to be 30.1 ± 9.8 pmol/mg/h, reaching an endpoint of 650±51 pmol/mg protein, or approximately 19–24 μM in the cell. For efflux, hBMVEC were loaded with 20 μM ^14^C-PBT434 at 37°C for 30min and then incubated in efflux medium at 37°C for 0–2.5h, and the amount of ^14^C-PBT434 remaining in lysates at each time point was plotted, n = 3–4 experimental replicates/time point (**B**). The line is a standard exponential fit to the data. The apparent plateau estimated by this fit is 27±4 pmol/mg protein, or ~0.5–0.6 μM remaining in the cells.

PBT434 is an orally bioavailable drug that can readily penetrate the BBB, as seen in studies carried out in mice and humans [30, 38, 39]. We monitored the accumulation of PBT434 in hBMVEC grown in monolayers using ^14^C-labeled PBT434 as a radiotracer. The data indicated that in a first phase ^14^C-PBT434 rapidly equilibrated between the uptake medium and the cell. This initial uptake was followed by an additional slow accumulation over 3 h which exhibited a rate of 30.1 ± 9.8 pmol/mg/h (**Fig 3A**). In the uptake protocol, uptake is quenched and cells washed at 4°C prior to processing for ^14^C-PBT434 accumulation (Methods). In a separate experiment, we examined the efflux of ^14^C-PBT434 from hBMVEC after a 30 min loading period. In the efflux protocol, cells are washed at 25°C. The data in **Fig 3B** indicate that in the 25°C wash approximately 92% of the cell-accumulated ^14^C-PBT434 was lost (*cf* 550 pmol ^14^C-PBT434/mg protein in **3A** at 30 min to 43 pmol ^14^C-PBT434/mg protein at t = 0 in **3B**). There was a further slow loss of the remaining ^14^C-PBT434 (**Fig 3B**). The data suggest two aspects of the accumulation and efflux of PBT434 by hBMVEC. Flux across the plasma membrane is rapid reaching what appears to be an equilibrium whether during uptake or efflux. However, in both processes there appears another slower process. This suggests that within the cell, some fraction of cell PBT434 is in a locale/state that is in a kinetic steady-state relationship with the fraction in equilibrium with the extra-cellular milieu. The kinetic analysis noted in **Fig 3B** estimated this pool of PBT434 was represented by 27±4 pmol/mg protein in the cell lysate when cells were treated with 20 μM reagent.

**Fig 4:**
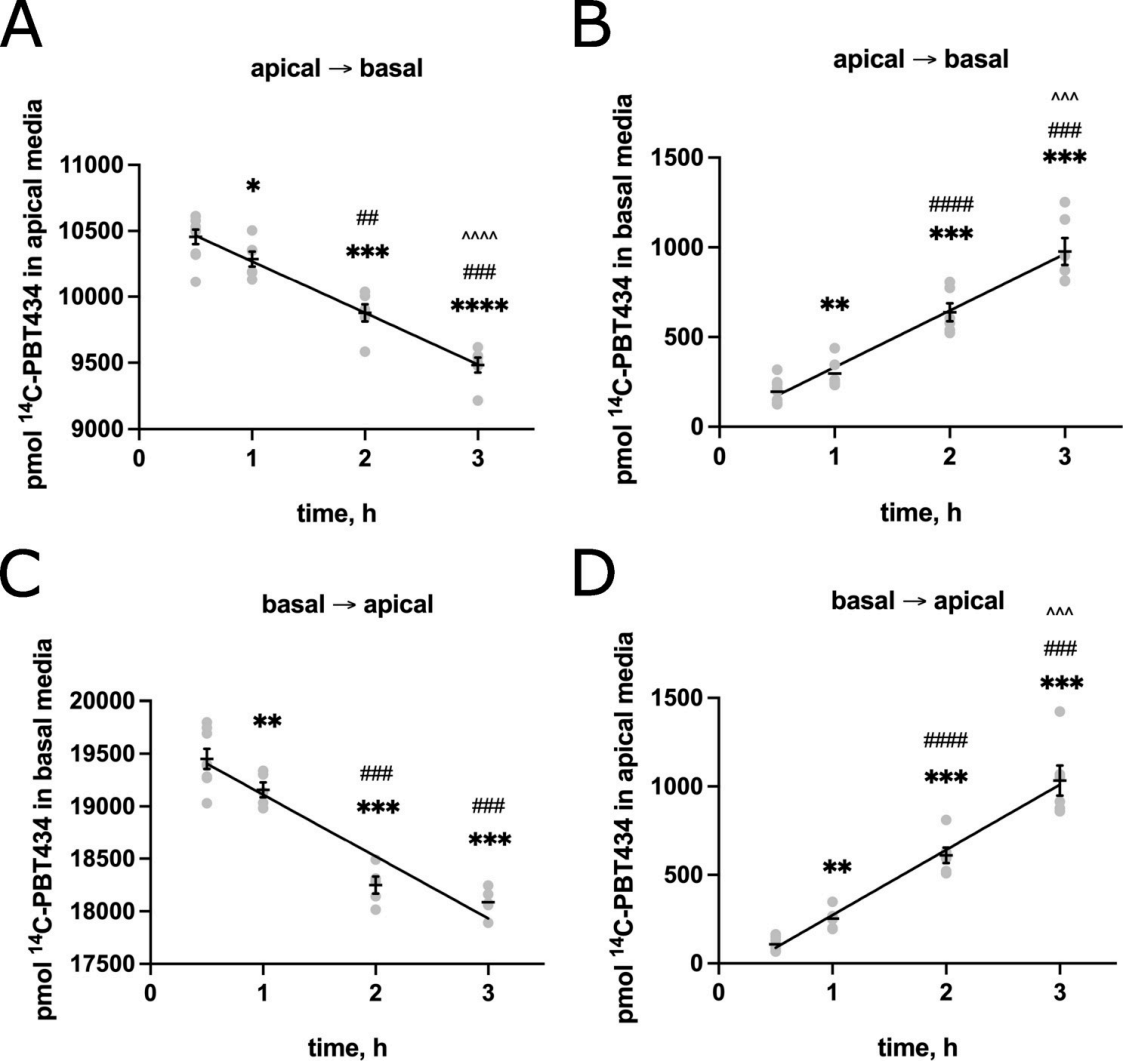
^14^C-PBT434 fluxes across an hBMVEC transwell model of the blood-brain barrier. hBMVEC grown in the apical chamber of Transwell Thincerts™ were loaded with 20 μM ^14^C-PBT434 in the apical chamber, and the mass of ^14^C-PBT434 in both the apical (**A**) and basal **(B**) chamber was quantified over 3h. Data are represented as mean ± SEM, n = 6–9 experimental replicates/time point. Statistical significance was determined using one-way ANOVA and Tukey’s multiple comparison test. *, statistically significantly different from 0.5h; **, p < 0.01; ***, p < 0.001; ****, p < 0.0001; #, difference from 1h; #, p < 0.05; ###, p < 0.001; ####, p < 0.0001; ^, difference from 2h; ^^, p < 0.01; ^^^^, p < 0.0001. hBMVEC grown in the apical chamber of Transwell Thincerts™ were loaded with 20 μM ^14^C-PBT434 in the basal chamber, and the mass of ^14^C-PBT434 in both the apical (**C**) and basal (**D**) chamber was quantified over 3h. Data are represented as mean ± SEM, n = 4–9 experimental replicates/time point. Statistical significance was determined using one-way ANOVA and Tukey’s multiple comparison test. *, statistically significantly different from 0.5h; **, p < 0.01; ***, p < 0.001; ****, p < 0.0001; #, difference from 1h; #, p < 0.05; ###, p < 0.001; ####, p < 0.0001; ^, difference from 2h; ^^, p < 0.01; ^^^^, p < 0.0001. Rates of ^14^C-PBT434 uptake or efflux were calculated using linear regression analysis. Note: the volume of the apical media is 0.5 ml compared to 1 ml of basal media.

To examine the transcellular flux of PBT434, we employed a well-validated *in vitro* BBB model using hBMVEC grown on the apical side of a transwell membrane [35, 36, 40, 41]. The barrier properties of these transwell cultures were verified by quantification of their transendothelial electrical resistance (TEER) and impermeability to FITC-labelled dextran (**S1 Fig**). We compared ^14^C-PBT434 uptake at the luminal (or apical, blood side) (**Fig 4A**) to uptake at the abluminal (or basolateral, brain side) (**Fig 4C**) membrane. In the same experiment, the corresponding efflux (transcellular flux) was quantified by the appearance of ^14^C-PBT434 in the efflux chamber (**Fig 4** panels **B** and **D**). The rates of these processes are provided in **Table 1**. The *mass* data illustrated in **Fig 4** (panels **B** and **D**) show that the *net* flux of PBT434 across this model blood-brain barrier was the same in the two directions. There were 976±185 pmol ^14^C-PBT434 accumulated in the basal chamber (**Fig 4B**) and 1033±210 pmol quantified in the basal chamber (**Fig 4D**). This near equivalence was reflected also in the closely similar rates of PBT434 *efflux* at the two barrier membranes (**Table 1**). However, there was a significantly greater *uptake* of PBT434 at the basolateral membrane in this barrier model as illustrated by the ~50% greater loss of compound from the basal chamber (**Fig 4C**) that corresponded to a ~40% greater rate of *apparent* cell uptake (**Table 1**). A more robust uptake would be predicted to result in greater accumulation. In fact, analysis of the cells at 3h showed that they retained ~6 μM PBT434 irrespective of the flux direction. The values were 8.1±1.3 μM (apical to basal) and 4.7±1.2 μM (basal to apical). As noted above, this analysis follows washing of the cells prior to lysis and quantification of total cell protein and ^14^C-PBT434. In addition, the medium in the apical chamber contained RPMI *plus* 10% FBS and 10% NuSerum whereas the basal, ‘brain’ chamber contained only RPMI (Methods). A reasonable inference was that the apparently greater ‘uptake’ at the basal membrane reflected a cell surface adsorption of PBT434 that was limited in the apical chamber by the presence of protein components in the serum. Upon washing the cells for *accumulation* of PBT434, this adsorbed material (that registered as ‘uptake’) was removed. Repeating this flux experiment but with serum in the basal chamber demonstrated that, indeed, serum suppressed this likely cell surface PBT434 adsorption (**S2 Fig**).

**Table 1:** Rates corresponding to fitted lines in Fig 4.

Flux Trajectory, process	Rate, pmol/h (±SD)
Apical → Basal, uptake	391 ± 30^a^
Basal → Apical, uptake	590 ± 54^a^
Apical → Basal, efflux	316 ± 23^b^
Basal → Apical, efflux	369 ± 22^b^

^a, b^ Significantly different at p<0.0001. Values from fit of data in Fig 4 to standard exponential (Prism 8).

### PBT434, unlike bipyridyl, does not limit the intracellular availability of labile iron

**Fig 5:**
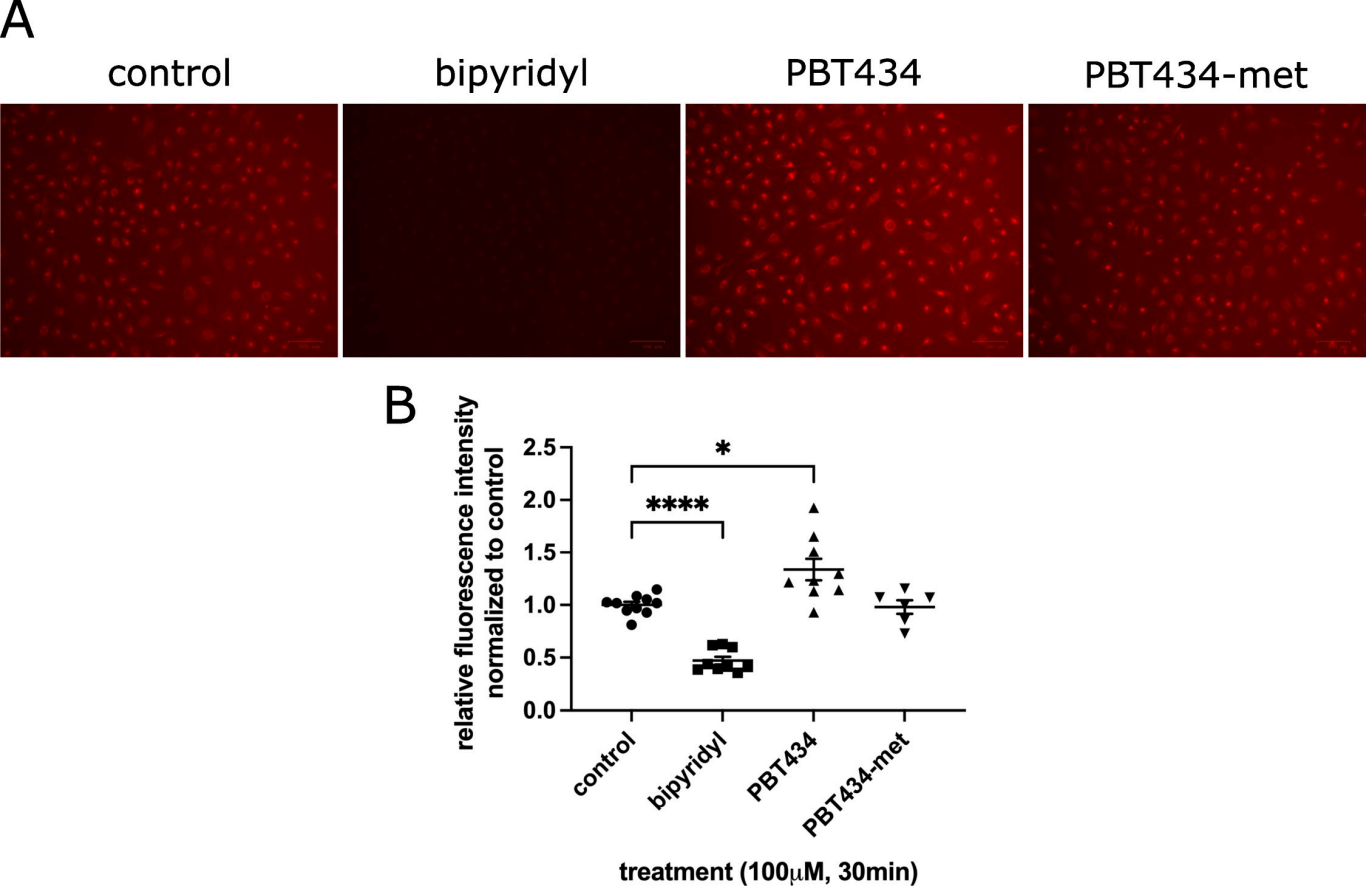
PBT434 increases intracellular iron staining, while bipyridyl severely limits the intracellular labile iron pool. hBMVEC treated with 100 μM of bipyridyl, PBT434 or PBT434-met (structures shown in Fig 1A and 1B) in combination with 1 μM FerroOrange for 30 min, after which live cells were imaged (A) and the total fluorescence intensity was quantified (B). Data are represented as mean ± SEM, n = 5–10 biological replicates. Statistical significance was determined using Welch’s ANOVA with Dunnett’s T3 multiple comparison test to compare means. *, statistically significant compared to control; *, p < 0.05; ****, p < 0.0001.

Since PBT434 has a more moderate affinity for iron compared to classical iron chelators such as deferiprone or bipyridyl, we examined how that difference was reflected in the PBT434 effect on the cellular labile iron pool (LIP) of hBMVEC. To do this, we took advantage of the permeable, Fe^2+^-specific fluorescent dye FerroOrange, which reacts with chelatable cytoplasmic iron. We saw a significant ablation of fluorescence in cells when treated with bipyridyl, consistent with chelation of the LIP by this high-affinity ferrous iron chelator and thus blocking the action of the fluorescent iron indicator (**Fig 5A**). In contrast, PBT434 did not compete with FerroOrange for Fe^2+^, a behavior consistent with its more moderate affinity [30]. In fact, the results showed that PBT434, but not the PBT434-met inactive derivative, induced a 34 ± 9% increase in FerroOrange-accessible Fe^2+^ suggesting this chelating agent mobilized iron within the cell without concurrent toxicity. Data presented below suggests this iron came from ferritin.

**Fig 6:**
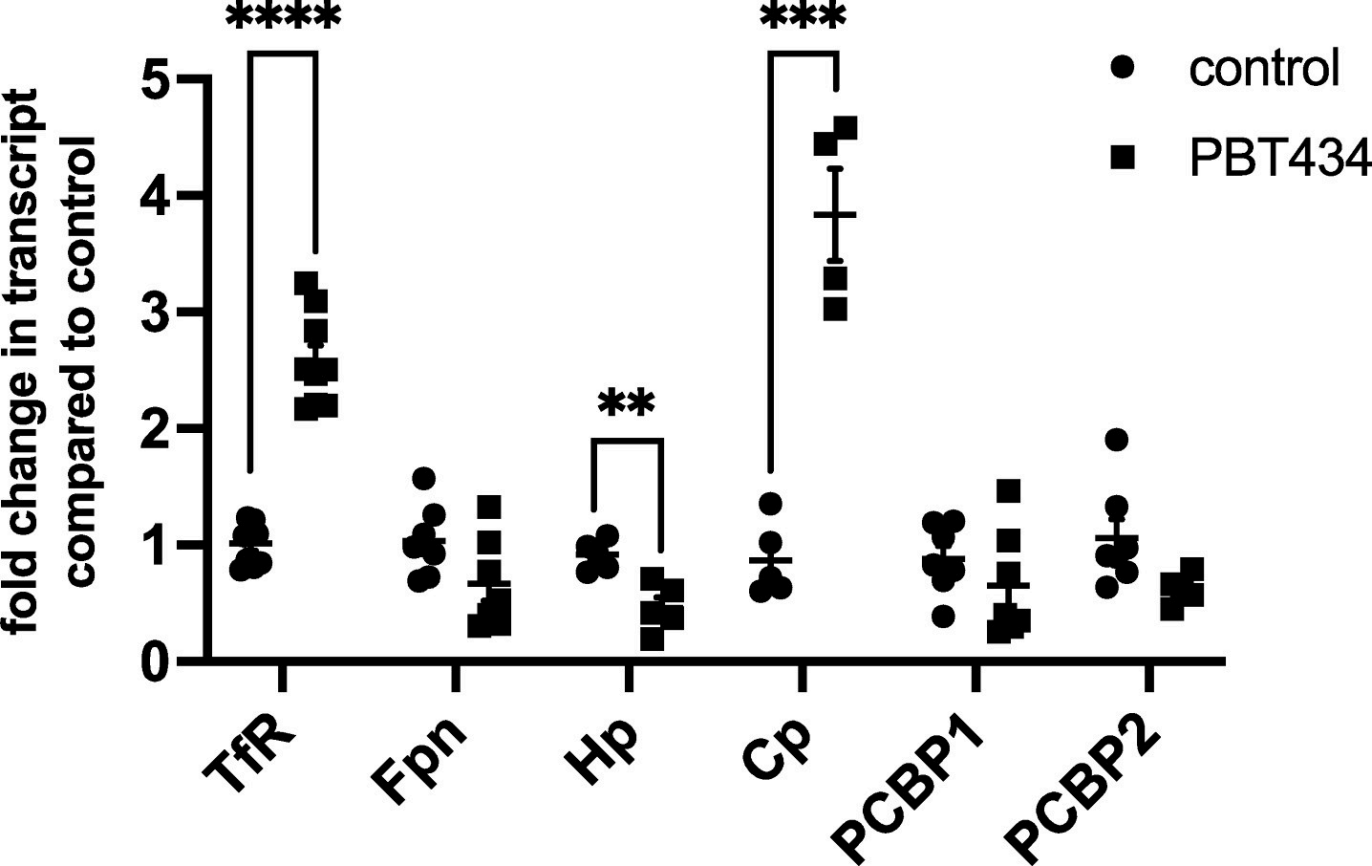
qPCR analysis of transcript abundance in PBT434-treated hBMVEC. hBMVEC were treated with 20 μM PBT434 or PBT434-met for 24h. RNA was collected and reverse-transcribed, and the resulting cDNA was quantified by qPCR. Transcript abundance in each sample was normalized to β-actin transcript, and the results are represented as the mean ± SEM fold change relative to control cells, n = 4–8 samples/condition. Statistical significance was determined by unpaired t-test between control and treated cells for each transcript; ****, p < 0.001 compared to control.

PBT434 was shown previously to restore depleted ferroportin protein expression in MPTP-treated mice to a level similar to that of unlesioned mice [30]. This result, along with the increase in intracellular ferrous iron staining in response to PBT434, suggested a potential effect on the cellular iron response system and function of downstream iron-related proteins. To assess this, we first conducted quantitative PCR (qPCR) analysis of the PBT434 effect on the abundance of the transcripts for several iron-handling proteins (**Fig 6**). While the transcripts for the iron efflux protein, ferroportin (Fpn) and the two cytoplasmic iron chaperones, PCBP1 and 2 were unaffected, the abundance of the mRNAs for the transferrin receptor (TfR), and the ferroxidase, ceruloplasmin (Cp), did change. TfR and Cp transcripts increased by 2.8 and 3.6-fold, respectively. Transferrin receptor (TfR) expression is linked to the iron-responsive element (IRE)/iron regulatory protein (IRP) system [42–44]. The increase in TfR mRNA suggests that PBT434 competes with the PCBP1-dependent delivery of iron for the assembly of the Fe,S cluster that converts the regulatory IREBP from an RNA binding protein to cytosolic aconitase [45]. Thus, PBT434 shifts this regulatory modulation towards RNA-binding and the corresponding inhibition of TfR mRNA degradation. In cell iron deficiency, Cp expression is, in part, regulated by HIF-1α [46]. Increase in HIF-1α function follows from the knock-down of its hydroxylation by prolyl hydroxylase activity in an iron-dependent reaction [47]. As in the case of the IREBP, PBT434 appears to diminish the pool of iron that serves as co-factor in HIF-1α hydroxylation and degradation. In this model, the increase in the steady-state level of this transcriptional activator increases Cp transcription.

**Fig 7:**
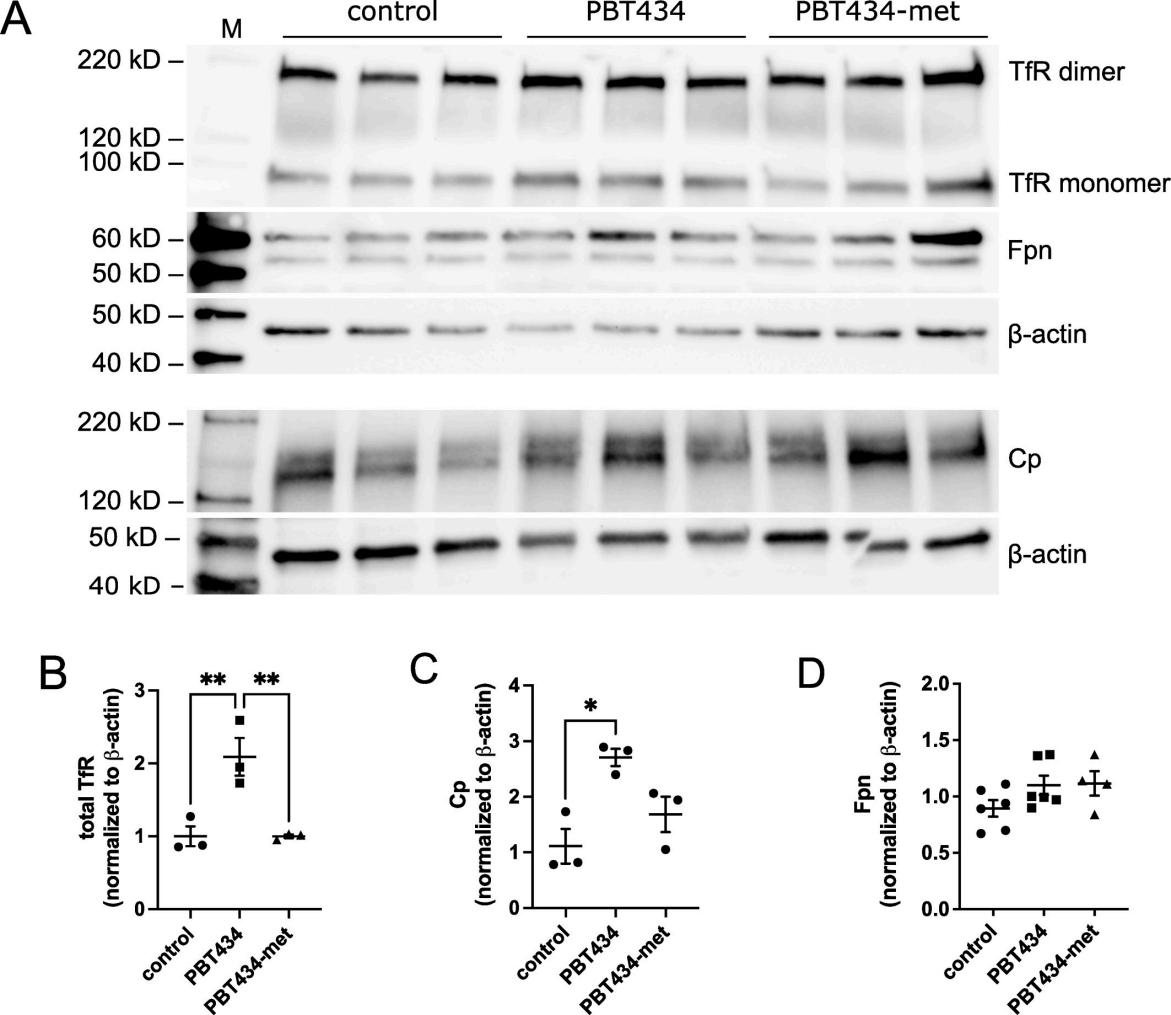
Western blot analysis of iron metabolic protein abundance in PBT434-treated hBMVEC. hBMVEC were treated with 20 μM PBT434 or PBT434-met for 24h and then cells were processed for Western blotting (**A**). Blots were probed for either anti-Fpn, anti-TfR, or anti-Cp, and anti-β-actin as a loading control. Band intensities were normalized to β-actin, and quantification of the relative band densities are given in panels (**B**-**D**). Data are represented as mean ± SEM, n = 3–6 biological replicates. Statistical significance was tested using one-way ANOVA and Tukey’s multiple comparison test; *, p < 0.05, **, p < 0.01 compared to indicated group.

Using a combination of ELISA analysis and western blotting, we probed the expression of iron-handling proteins in PBT434 or PBT434-met treated hBMVEC; examples of the WB analyses are given in **Fig 7A**. The data showed the abundance of TfR monomer and dimer were significantly increased by 24h as was Cp (**Fig 7B** and **7C).** Both increases paralleled the PBT434-dependent increase in the respective transcripts (**Fig 6**), In contrast, the expression of the iron efflux protein, Fpn, was insensitive to PBT434 treatment (**Fig 7D**).

**Fig 8:**
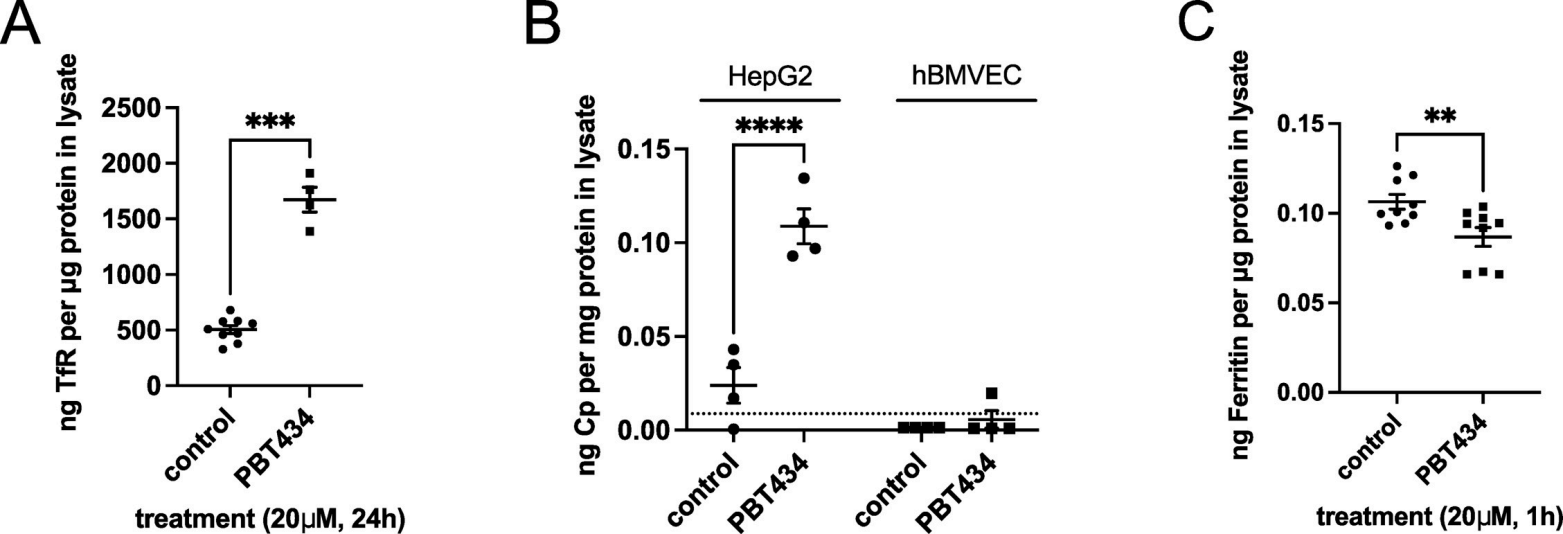
ELISA analysis of iron metabolic protein abundance in PBT434-treated hBMVEC. **(A**) hBMVEC were incubated in the absence or presence of 20 μM PBT434 for 24h. Cell lysates were assayed for transferrin receptor (TfR) by sandwich ELISA and normalized to total protein content. Data are represented as mean ± SEM, n = 4–9 biological replicates. Statistical significance was tested using t-test; ****, p < 0.0001, compared to control. The mean values were: 505 ± 109 (control) and 1672 ± 223 ng TfR/μg total cell protein (+PBT434). (**B**) hBMVEC or HepG2 were grown in monolayers for 24h in media containing serum, then incubated in the absence or presence of 20 μM PBT434 in media without serum for an additional 24h. Following incubation, cells were treated with PI-PLC (0.5 U/ml) for 1h to release any GPI-anchored cell surface proteins. Media was collected, concentrated, assayed for Cp protein by sandwich ELISA and normalized to total protein content. Data are represented as mean ± SEM, n = 4 biological replicates. Statistical significance was tested using t-test; ****, p < 0.0001, compared to control. The lower sensitivity limit of this kit is reported to be 0.12 ng/ml (or 0.0088 ng/mg protein average) indicated by the dashed line. (**C**) hBMVEC were loaded with 1 μM Fe^2+^-citrate in RPMI+serum media for 24h, then washed and incubated in the absence or presence of 20 μM PBT434 for an additional 1h. Cell lysates were assayed for ferritin protein by sandwich ELISA and normalized to total protein content. Data are represented as mean ± SEM, n = 9 biological replicates. Statistical significance was tested using t-test; *, p < 0.05, compared to control. The mean values were: 106 ±4 (control) and 87 ± 5 pg Ft/μg total cell protein (+PBT434).

We used ELISA as an additional method to quantify the fold-changes indicated by the western blot data. Thus, hBMVEC were treated with PBT434 for 24h and cell lysates were assayed by ELISA for TfR (**Fig 8A**). The fold-increase in TfR in response to PBT434 treatment quantified by ELISA was equivalent to that provided by analysis of the western blots (**Fig 7B**). ELISA was used also to assess secreted and GPI-linked Cp protein abundance, using HepG2 cells as a positive control. With respect to Cp secreted into growth media, this approach was limited in that sCp abundance in both HepG2 and hBMVEC conditioned media was at or below the lower sensitivity limit of this assay (**S3 Fig**). However, it did enable the assessment of GPI-Cp abundance. In this method, cells were treated with phosphatidylinositol-specific phospholipase C (PI-PLC), which cleaves the GPI anchor; the media thus conditioned was concentrated and analyzed by the Cp-ELISA. While this approach demonstrated PBT434 increased the amount of GPI-Cp in HepG2 cells, it again failed to detect any Cp released by the PI-PLC (**Fig 8B**). ELISA also afforded a direct method for quantification of ferritin. To do so, hBMVEC were loaded with 1 uM Fe-citrate for 24h, followed by treatment in the absence or presence of PBT434 for an additional 1 h. The resulting cell lysates were subjected to ELISA analysis for ferritin (**Fig 8C**). In contrast to the increase in TfR, treatment with PBT434 knocked down ferritin (Ft) protein by ~18%. Indeed, this loss of Ft protein was apparent after only 1h treatment with the reagent. The temporal nature of this result can be correlated with the increase in chelatable Fe^2+^ noted above following a 30 min treatment with PBT434. As discussed later, knock down of ferritin has been demonstrated following treatment with other cell-permeant Fe^2+^ chelating agents [48].

### ^55^Fe^2+^ uptake is inhibited by complexation with PBT434

**Fig 9:**
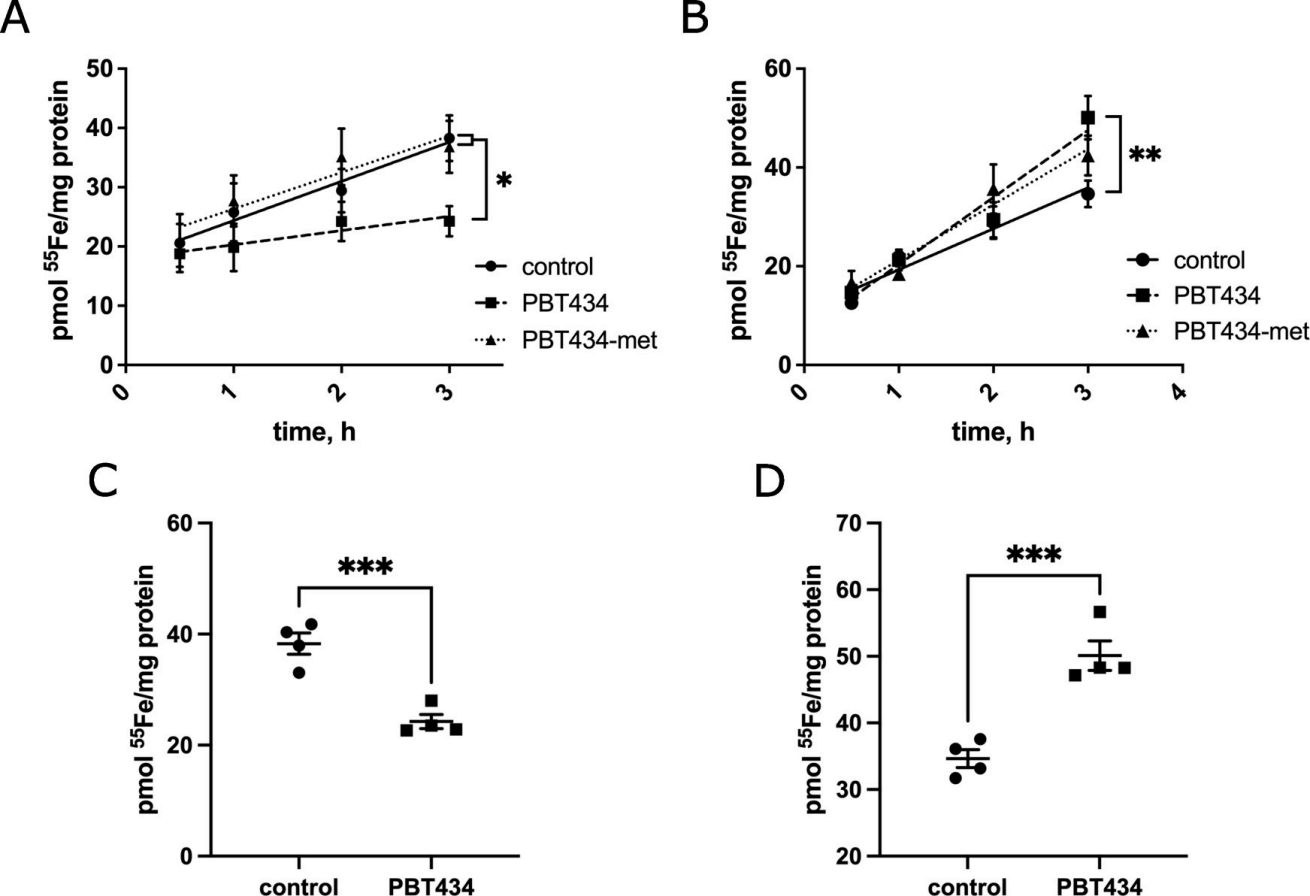
^55^Fe^2+^ uptake by hBMVEC is inhibited by complexing with PBT434, but not with PBT434 pre-treatment. hBMVEC were loaded with 1 μM ^55^Fe^2+^ at 37°C for 3h in cells in complex with 20 μM PBT434 or PBT434-met in the loading media (**A-C**) or pre-treated with PBT434 or PBT434-met for 24h prior to the start of uptake (**B-D**). Cells were lysed over the indicated period of time and the amount of ^55^Fe^2+^ accumulated at each timepoint (**A, B**) and after 3h (**C, D**) was calculated. Data are represented as mean ± SEM, n = 4 experimental replicates/time point. The rates of ^55^Fe^2+^ accumulation between 0.5-3h were determined using linear regression analysis. Statistical significance was determined using one-way ANOVA and Tukey’s multiple comparison test. *, statistically significantly different from control or as indicated; *, p < 0.05; **, p < 0.01; ***, p < 0.001.

Given the rapid equilibration of PBT434 in hBMVEC within 30 min, in comparison to the slow, biphasic uptake and equilibration of Fe^2+^ over 24h [49], we hypothesized that PBT434 and Fe^2+^ did not share the same uptake mechanism. To test this, hBMVEC monolayers were incubated with radiolabeled ^55^Fe^2+^ in the absence or presence of PBT434 or PBT434-met, and ^55^Fe^2+^ uptake over 3 h was monitored (**Fig 9A**). PBT434 significantly decreased the rate of ^55^Fe^2+^ uptake, as well as decreased the overall accumulation of ^55^Fe^2+^ in cell lysates (**Fig 9C**). This effect was not seen with PBT434-met. Comparison of PBT434 to ^55^Fe-uptake rates indicate that PBT434 and Fe^2+^ are taken up by separate transport pathways. Furthermore, the inhibition of ^55^Fe-uptake in the presence of PBT434 but not PBT434-met suggests that an extra-cellular PBT434-iron complex is not a ligand for the ferrous iron transporters in hBMVEC, namely ZIP8 and ZIP14.

To further examine the role of PBT434 in iron accumulation, we tested the effect its pre-exposure had on ^55^Fe^2+^ uptake. Cells pre-treated with PBT434 which were, after washing, exposed to ^55^Fe^2+^ displayed an increase in the rate of uptake and accumulation of ^55^Fe^2+^ after 3h (**Fig 9,** panels **B and D**). This increased accumulation was maintained for at least 24h (**S4 Fig**). These data suggest that pre-exposure of cells to PBT434 transiently potentiates iron uptake. Unexpectedly, PBT434-met pre-treatment also showed an increase in both uptake and accumulation (**Fig 9B**), but this effect was not as significant or persistent as that shown by PBT434 (**S4 Fig**).

**Fig 10:**
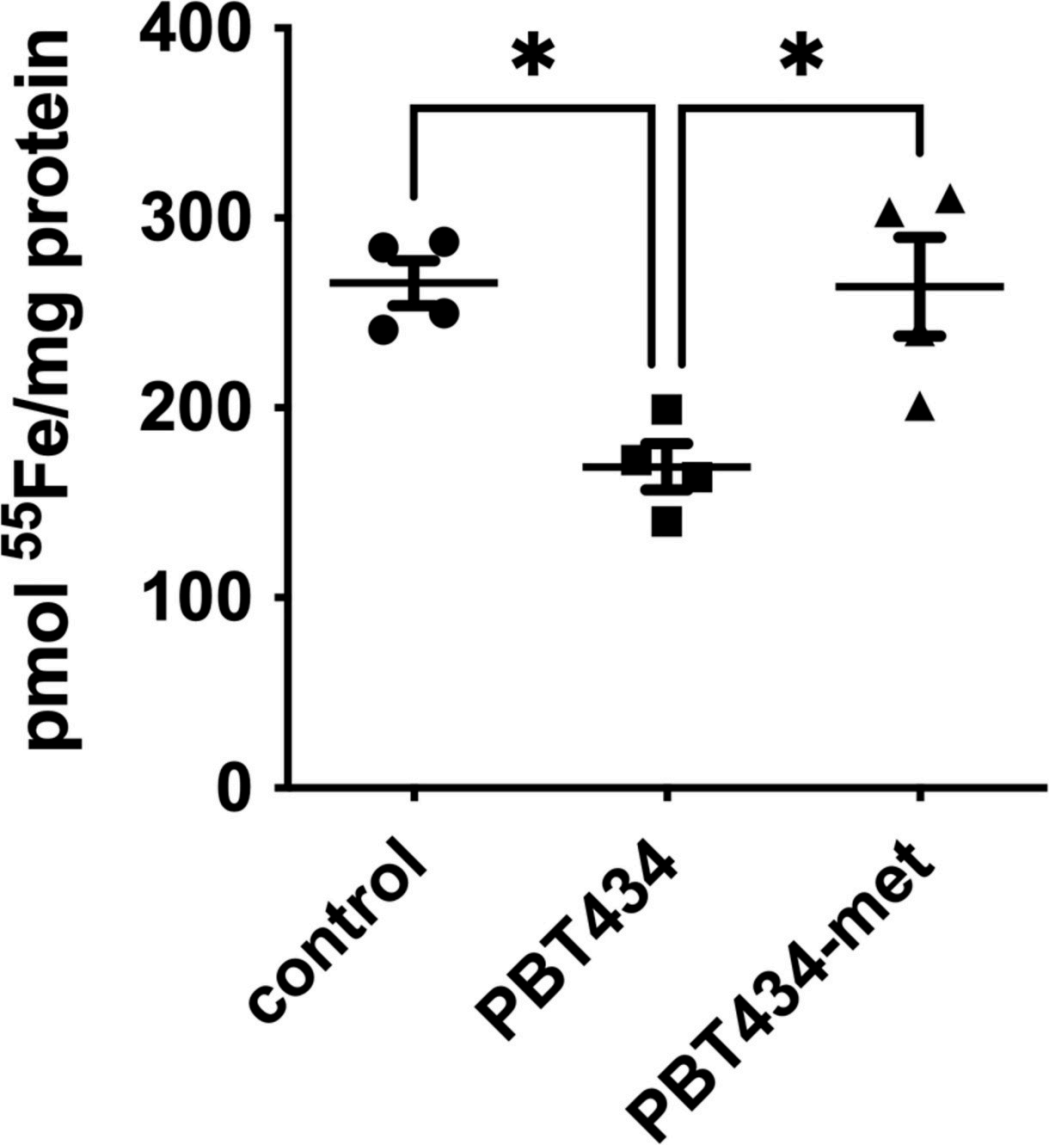
^55^Fe^2+^-transferrin uptake by hBMVEC is inhibited by complexing with PBT434. hBMVEC were loaded with 1 μM ^55^Fe^2+^-Tf at 37°C for 3h in cells in complex with 20 μM PBT434 or PBT434-met in the loading media. After 3h, cells were lysed and the amount of ^55^Fe^2+^ accumulated was calculated. Data are represented as mean ± SEM, n = 4 experimental replicates. Statistical significance was determined using one-way ANOVA and Tukey’s multiple comparison test. *, statistically significantly different from control or as indicated; *, p < 0.05.

We have shown that uptake of iron from ^59^Fe-transferrin is supported by ferri-reduction and ferro-permeation at the plasma membrane of hBMVEC [50, 51]. One experimental outcome in support of this TBI iron uptake model was knockdown of this uptake by inhibition of extra-cytoplasmic ferrireductase activity; another result was a 60% inhibition of TBI iron uptake by ferrozine, a strong ferrous iron chelating agent [50]. This latter strategy was utilized to demonstrate that PBT434, but not PBT434-met, also inhibited TBI iron uptake (**Fig 10**).

### PBT434 stimulates Fpn-dependent ^55^Fe^2+^ efflux

**Fig 11:**
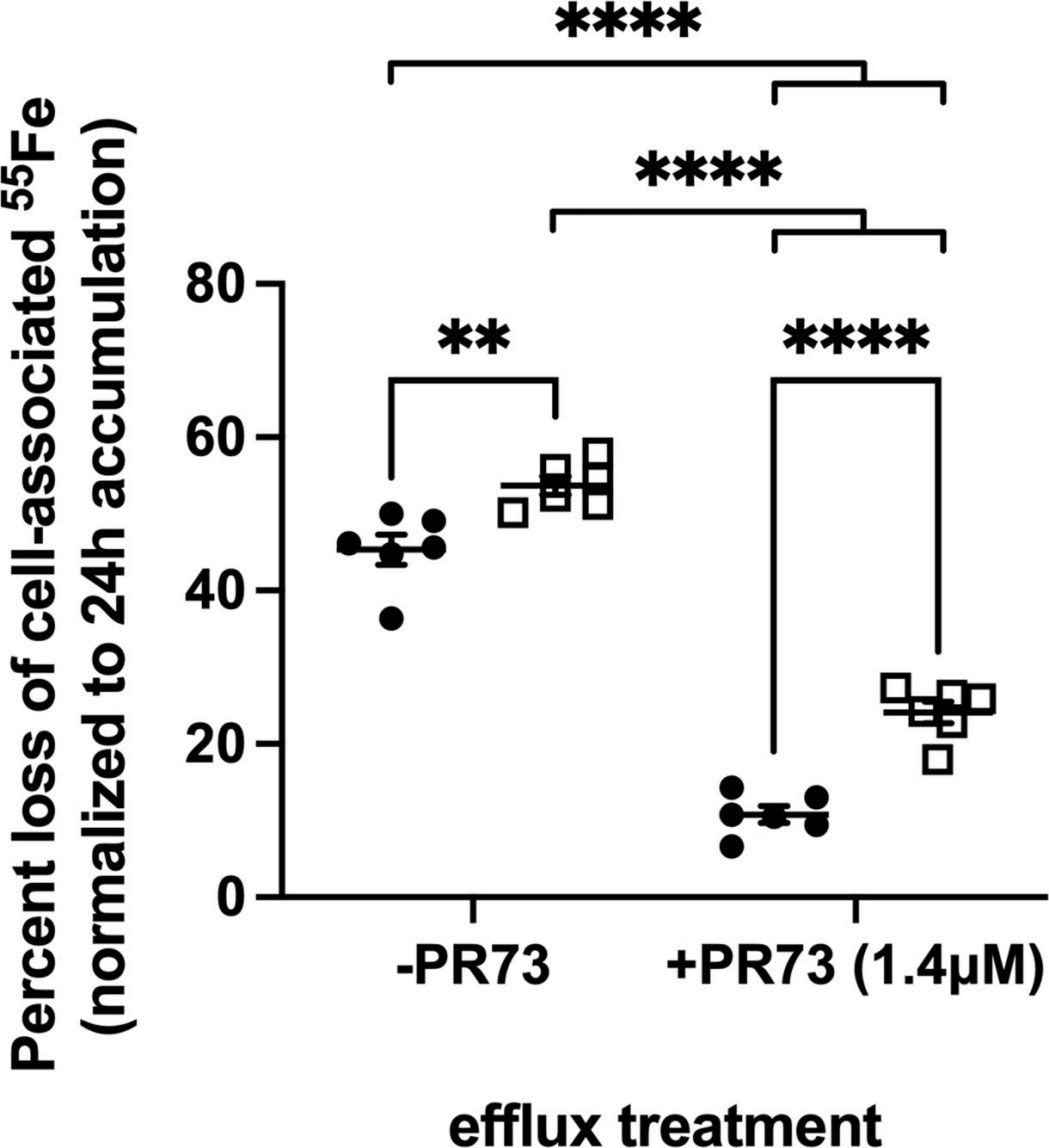
PBT434 stimulates ferroportin-dependent ^55^Fe^2+^ efflux. hBMVEC were loaded with 1 μM ^55^Fe^2+^ at 37°C for 24h in the absence (-PR73) or presence of mini-hepcidin PR73 (+PR73, 1.4 μM). Iron efflux was then initiated in efflux medium alone (control, closed circles), or media containing PBT434 (20 μM, open squares). The amount of ^55^Fe lost from the cells after 6h incubation was quantified; the data are presented as the percent loss of the initial cell-associated ^55^Fe^2+^. Data are represented as mean ± SEM, n = 6 biological replicates/condition. Statistical significance was determined using two-way ANOVA and Tukey’s multiple comparison test. **, p < 0.01; ****, p < 0.0001 compared to control or as indicated.

PBT434 has approximately 20% the ability of deferiprone to produce an apparent stimulation of Fe^2+^ efflux from neuronal cells [30]. We assessed the efflux of ^55^Fe^2+^ from hBMVEC in the absence or presence of PBT434 in control cells or cells treated with a mini-hepcidin, PR73. Hepcidin is a peptide hormone found both systemically and in the brain interstitium that binds to Fpn and targets the transporter for degradation. The effects of hepcidin on the iron export function of Fpn have been extensively studied [52–54]. We have previously shown efflux of Fe^2+^ from hBMVEC is Fpn-dependent [35, 49]. PR73 has an EC_50_ of ~4 nM for Fpn degradation in a GFP reporter assay [55]. hBMVEC in monolayers were loaded with ^55^Fe^2+^ for 24 h in the absence or presence of PR73. ^55^Fe-efflux was then quantified over a 5h period in the continued absence or presence of PR73 in combination with the absence and presence of PBT434 (**Fig 11**). While PR73 knocked down ^55^Fe efflux from both the control and PBT434-treated cultures, PBT434 partially suppressed the inhibition due to the mini-hepcidin. In the absence of PBT434, iron efflux from the PR73-treated cultures was knocked down by ~75% while knock down in the PBT434-treated cultures was only ~50% (**Fig 11** and **Table 2**). Two inferences can be drawn from these results. First, knockdown of Fpn by PR73 down-regulates ^55^Fe-efflux in the presence as well as the absence of PBT434. Second, under either condition, PBT434 supports a significant albeit small stimulation of iron efflux.

**Table 2:** hBMVEC ^55^Fe-efflux (data from Fig 11).

Cell sample	Percent ^55^Fe loss (±SD)
Control, -PBT434	45.3 ± 4.9
Control, +PBT434	53.7 ± 3.0
+PR73, -PBT434	10.8 ± 2.7
+PR73, +PBT434	24.1 ± 3.4

## Discussion

Due to the moderate iron-binding affinity of PBT434, this novel compound is being investigated as a potential therapeutic iron chelator in neurodegenerative disorders characterized by increased iron levels in areas associated with neuropathology [30, 31, 38, 39, 56]. PBT434 has been well-tolerated in an early phase clinical trial in healthy volunteers but understanding whether this drug disrupts normal iron homeostatic mechanisms is imperative for its clinical use. In this study, we examined the effects of this drug on physiologic iron trafficking using an *in vitro* hBMVEC blood-brain barrier model. Our data show that binding of Fe^2+^ by PBT434 limits transport of ^55^Fe^2+^ by the ferrous iron uptake proteins, has a moderate ability to alter the labile iron pool and stimulates iron efflux. The compound itself readily equilibrates across the hBMVEC BBB model used here and thus would have comparable impacts on iron handling within the interstitial space.

The use of metal chelation therapy for treatment of neurodegenerative diseases has been a promising area of study, although many of the original chelators and their successors have presented problems due to issues with efficacy or severe adverse effects [2, 3, 57–60]. Namely, high affinity chelators, while able to scavenge excess iron and prevent oxidative stress-induced degeneration, tend to pull iron out of iron-dependent proteins that are necessary for cellular function. Since PBT434 has a more moderate iron affinity, it was predicted to sample the labile iron pool without blocking the utilization of this iron for assembly of iron-containing prosthetic groups, *i*.*e*., Fe,S clusters and metallated porphyrins. Indeed, our data show that treatment with supra-physiologic concentrations of PBT434 for 1 h increased both the intracellular labile iron pool, an increase that correlated with a turnover of ferritin. In contrast, treatment for 24 h led to an increased expression of iron-regulated proteins including the transferrin receptor, TfR and the ferroxidase ceruloplasmin, a protein involved in iron efflux. The increase in expression of TfR *via* the canonical 3’ untranslated region IRE provides evidence for an iron-restricted state in as much stabilization of the TfR mRNA by the IREBP occurs when the latter lacks its Fe,S cluster. The increase in FerroOrange detectable Fe^2+^ and this apparent iron-restricted state appear in conflict. However, this pattern is consistent with the model that PBT434 competes with the cytoplasmic iron chaperones, PCBP1/2, in their support of cytoplasmic Fe,S cluster assembly. By blocking iron chaperone function, PBT434 prevents that ‘turn off’ of the mRNA binding activity of the IREBP. Further experiments investigating the impact of PBT434 specifically on the cytoplasmic IRE/IREBP regulatory system are ongoing.

The rapid knock down of Ft following PBT434 treatment was unexpected, although it did correlate with the increase in chelatable Fe^2+^. Ft turnover is mediated by both the lysosome and proteasome [61–64]. There is fair agreement as to the signals that target Ft to one or the other pathway, there is less consensus as to the temporal relationship between iron release and degradation. Not surprisingly, data are clear that degradation does result in iron release; less thoroughly examined in a cell context is the release of iron upstream of protein turnover. One such study in HEK293T cells demonstrated a temporally identical Ft knock down following treatment with the ferric iron chelators, deferoxamine, desferasirox and deferiprone [65]. A similar result was reported in a hepatoma cell line treated with desferasirox [66]. However, none of these studies probed for coincident appearance of chelatable ferrous iron as we have reported here. Additional studies on the mechanism of PBT434 ferritin iron release and turnover are in progress.

Importantly, therapeutic iron chelators must readily cross the blood-brain barrier and reach a sufficient concentration in the brain interstitium. In this study, we examined the trafficking of PBT434 across an *in vitro* BBB model and its effects on the release and re-uptake of iron. We used 20 μM PBT434 for most experiments, as this corresponds to the highest concentration of the drug used in previous studies [30, 38, 39]. This is higher than the maximum cerebrospinal fluid (CSF) concentrations observed in Phase I clinical trials (approximately 150 ng/ml, or 0.5 μM [38, 39]), but our data show PBT434 is well tolerated in hBMVEC at significantly higher concentrations without any significant loss of metabolic activity. PBT434 readily accumulates in hBMVEC reaching an apparent equilibrium after 3h with a concentration of approximately 19–24 μM within the cell, roughly equivalent to the PBT434 concentration in the media.

In order to determine if PBT434 crosses a model BBB barrier, we grew hBMVEC in transwell inserts, allowed them to polarize and form tight junctions, and subsequently examined the trajectory of PBT434 across this cell barrier following its addition to either the apical or basolateral compartment. With an initial concentration of 20 μM PBT434 loaded in the apical chamber, in 3h ~1000 pmol PBT434 accumulated in the basal chamber to achieve a concentration of 1 μM. This represented ~5% of the compound present at the apical membrane. When PBT434 was loaded in the basal chamber, a similar quantity of compound accumulated in the apical chamber. While these behaviors apply only to the conditions of the experiment, *e*.*g*. the concentration of PBT434 and the media differences noted above, clearly a bi-directional flux of PBT434 across the BBB likely represents a significant aspect of its physiologic distribution and elimination.

One of the possible mechanisms of action for PBT434 is inhibition of iron-mediated protein aggregation *via* reduction of the labile iron pool. Interestingly, PBT434 increased staining of the intracellular LIP as indicated by FerroOrange imaging, as well as increased the expression of genes nominally responsive to cell iron status. This finding indicates there may be an alternate mechanism to explain the protective effects of PBT434. Indeed, our results indicate that the direct effect of iron-binding by PBT434 is to limit iron uptake, likely because the iron in the PBT434 complex is no longer a substrate for the ferrous iron transporters. In addition, we have shown that hBMVEC supports a plasma membrane ferri-reduction of transferrin-bound iron (TBI); indeed, inhibition of this ferri-reduction blocked TBI iron uptake [50]. This TBI-associated iron uptake was inhibited also in the presence of a ferrous iron chelator, ferrozine. These data indicate that TBI iron is reductively released from transferrin at the plasma membrane for subsequent Fe^2+^-uptake. Here we have shown that PBT434, like ferrozine, blocks hBMVEC uptake of ^55^Fe from ^55^Fe-Tf.

This model that PBT434 blocks hBMVEC iron-uptake by chelation of extracellular ferrous iron, whether from NTBI or TBI, would apply to the re-uptake of interstitial iron, a novel mechanism that has just recently been investigated for other transition metals at the BBB [36]. This inhibition of basolateral iron re-uptake by hBMVEC would result in an increased accumulation of iron in the abluminal space. That is, iron transported into the basal transwell chamber from hBMVEC is chelated by PBT434 and no longer is ligand for the iron uptake proteins present on the basolateral membrane. The metal binding capacities of several physiologic proteins are being investigated for this mechanism of inhibition of iron re-uptake within the interstitial space. Thus, soluble, metal-binding proteins such as α-synuclein, amyloid beta (Aβ), and secreted amyloid precursor protein (sAPP), can act as a biometal “sinks” to limit the cytotoxic effects of unbound metals in the brain interstitium [67–71]. Clearly, such interstitial iron chelators would knock-down iron uptake by all cells that access this compartment, not just BMVEC. While this interstitial chelation may be beneficial for mediating cell oxidative stress in the short term, the long-term accumulation of α-synuclein or Aβ and their associated metals, particularly in cases with causative genetic mutations or predisposing risks such as aging, could potentially drive the shift from normal physiology to pathology. In contrast, small molecule inhibitors such as deferiprone in PD patients [25] and PBT434 in murine PD and oxidative stress models [30] reduce pathology-induced increases in brain iron, with the latter potentially functioning extracellularly without an apparent accumulation of interstitial iron.

A key observation in these studies was the increase in ^55^Fe-efflux from cells treated with PBT434. Although the mechanism underlying this increase was not explicitly interrogated, one contributor to it very likely was the increase in FerroOrange-chelatable cytoplasmic ferrous iron. This is the substrate for the efflux transporter, ferroportin. Indeed, we demonstrated that ferroportin knockdown due to a mini-hepcidin, PR73, did suppress iron efflux from control as well as PBT434-treated cells. However, even in PR73 Fpn-knockdown cells PBT434 treatment supported a more robust efflux. These results indicate that while iron efflux in the presence of PBT434 does rely, at least in part, on ferroportin, the drug appears to suppress the PR73-dependent ferroportin removal from the plasma membrane. In this model, PBT434 serves both as a positive contributor to Fpn-mediated iron transport and a hepcidin antagonist. There are two facts which lend support to this latter inference. First, extracellular ferrous or ferric iron chelators have the potential to stabilize Fpn in the plasma membrane by scavenging exported iron, thus facilitating outward iron efflux [72]. Second, the recent solution of the Fpn structure shows that hepcidin binding to Fpn is dependent on Fe^2+^ bound at a solvent-accessible site in the transporter [73]. A small, amphipathic ferrous iron chelator like PBT434 could compete with hepcidin at that iron atom and potentiate its release into the extracellular milieu. Clearly, this model requires significant research for its validation, but does reflect basic features of the function and regulation of Fpn.

In summary, we provide *in vitro* evidence for the trafficking of PBT434 across the BBB and into the brain interstitium consistent with the results of early phase clinical trials. Additionally, we show that while PBT434 has moderate effects on the LIP and regulation of downstream iron-dependent protein expression, it does not significantly interrupt normal cell physiology, unlike high affinity iron chelators. In addition, PBT434 is able to bind and redistribute extracellular ionic Fe^2+^, limiting the downstream oxidative stress associated with this pro-oxidant and its role in cytotoxic protein aggregation. This novel mechanism of action provides a compelling case for the continued development of PBT434 as a therapeutic agent in neurodegenerative diseases correlated with metal accumulation.

## Materials and methods

### Reagents

PBT434, PBT434-met, and ^14^C-PBT434 were provided by Alterity Therapeutics (Melbourne, AUS) and reconstituted in DMSO. MTT (3-(4,5-Dimethylthiazol-2-yl)-2,5-Diphenyltetrazolium Bromide) was obtained from Invitrogen (Carlsbad, CA) and prepared fresh in PBS. Bipyridyl (2,2-bipyridine) was obtained from Sigma (St. Louis, MO) and reconstituted in DMSO. FerroOrange, an intracellular Fe^2+^ dye (ex, em: 543, 580nm), was obtained from Dojindo Molecular Technologies (Rockville, MD) and prepared in DMSO according to the manufacturer’s instructions. Phosphatidylinositol-specific phospholipase C (PI-PLC, Cat # P5542) was obtained from Sigma and resuspended at 100U/ml in 10mM Tris, 144mM NaCl, 0.05% BSA buffer, pH 7.4. ^55^Fe was obtained from Perkin-Elmer (Waltham, MA) as ferric chloride (NEZ04300). Mini-hepcidin (PR73) was a kind gift from Dr. Elizabeta Nemeth (UCLA); its use has been well characterized [52, 55].

### Cell culture

Human brain microvascular endothelial cells (hBMVEC) were a generous gift from Dr. Supriya Mahajan (University at Buffalo); the generation and characteristics of this line have been described in detail [34, 37]. HepG2 cells were obtained from Cell Applications (San Diego, CA) and cultured in RPMI growth media as described previously [35]. hBMVEC were cultured as previously described [50] to 90–95% confluence at the time of the experiment. RPMI1640 was supplemented with 10% FBS and 10% NuSerum (VWR) for RPMI+serum growth media or supplemented with 5 μg/ml human insulin and 30 nM Na selenite for RPMI-serum media. Experiments were performed in 35mm or 24-well tissue culture dishes, or 24-well ThinCert™ cell culture transwell insert (1.0 μm pore size, Greiner Bio-One). For transwell experiments, barrier integrity was measured by transendothelial electrical resistance (TEER) using a Millicell ERS-2 Epithelial Volt-Ohm Meter (EMD Millipore, Billerica, MA) and normalized to the surface area of the transwell (3.36cm^2^ for 24-well). The results are reported in **S1 Fig** and are comparable to those reported for this cell line previously [35].

### MTT assay

hBMVEC were grown to confluency in a 24-well plate, then treated with PBT434 at the indicated concentrations in cell culture media for 24h at 37°C. The next day, media was removed, and cells were incubated with RPMI+serum media containing MTT at 0.5mg/ml for 2h at 37°C, followed by incubation with 10% SDS/0.01N HCl for an additional 16h at 37°C to solubilize the MTT formazan crystals. Once solubilized, the solution was transferred to a 96-well plate in triplicates and the absorbance was read at 570nm on a plate reader (FLUOstar Omega, BMG Labtech, Cary. NC). Values were blank corrected and normalized to the untreated control. Cells treated with 0.1% Triton X-100 were used as a positive control for cell death.

### ^14^C-PBT434 accumulation and efflux assays

For ^14^C-PBT434 uptake, hBMVEC monolayers were loaded with 20 μM ^14^C-PBT434 in RPMI1640 plus serum growth media for up to 3h at 37°C. Reactions were quenched with ice-cold quench buffer, as previously described [50], and lysed in lysis buffer. The lysates were assayed for ^14^C counts (Beckman LS6500 Scintillation Counter) and normalized to protein content determined by BCA assay (Thermo Scientific, Waltham, MA).

For ^14^C-PBT434 efflux, hBMVEC monolayers were loaded with 20 μM ^14^C-PBT434 in RPMI plus serum growth media for 30min at 37°C, then washed twice with pre-warmed RPMI plus citrate and incubated in RPMI plus serum efflux media for an additional 2.5h. Every 30min, cells were quenched with ice-cold quench buffer, lysed, and processed as above. Cell-associated ^14^C counts were normalized to protein content.

For ^14^C-PBT434 trajectory assays, hBMVEC were grown in the apical chamber of transwell inserts were loaded with 20 μM ^14^C-PBT434 in either the apical chamber (RPMI+serum) or the basal chamber (RPMI-serum). Media samples were collected from both the apical and basal chamber at the indicated timepoints, and after 3h cells were quenched with ice-cold quench buffer, lysed, and processed the same as above. For a rough approximation of the intracellular PBT434 concentration at the endpoint of the uptake and efflux assays, the concentration was calculated from the pmol of ^14^C-PBT434 remaining in the cells using an estimation of 200K – 250K cells at confluence based on initial seeding density, and an approximated endothelial cell volume of 10,000 μm^3^ [74].

### FerroOrange imaging

hBMVEC were grown to confluence in a 24-well plate, washed twice with PBS (containing Ca^2+^, Mg^2+^), and then incubated in the presence or absence of 100 μM Fe^2+^ (ferrous ammonium sulfate, 250 μM citrate, 5 mM ascorbate) in RPMI+serum for 30min at 37°C. Cells were washed twice with PBS/Ca^2+^/Mg^2+^, then incubated with media containing 100 μM PBT434, PBT434-met, or bipyridyl and 1 μM FerroOrange) for an additional 30min at 37°C. Live cells were imaged using the ZOE fluorescent cell imager (Red Channel, Excitation: 556/20 nm, Emission: 615/61 nm) (Bio-Rad, Hercules, CA) and total fluorescence intensity was quantified using Fiji (Fiji Is Just ImageJ) software [75, 76]. Brightness of representative images was adjusted equally across all images using Fiji.

### RNA isolation and RT-qPCR

hBMVEC were grown to confluency in 35mm dishes, then treated with 20 μM PBT434 or PBT434-met for 24h at 37°C. Cells were washed twice with PBS and total RNA was isolated using TRIzol reagent (Invitrogen). RNA was purified using the Zymo Direct-zol RNA kit (Zymo Research), and 400 ng of RNA was reverse-transcribed using the qSCript cDNA synthesis kit (Quanta Bio, Beverly MA) to generate cDNA. Real-time quantitative qPCR was performed using 20 ng of cDNA with the iTaq™ Universal SYBR® Green Supermix and analyzed using a CFX96 Touch™ real-time PCR detection system (Bio-Rad). The amplification data were normalized to the mean of β-actin as a housekeeping gene, and the fold change in transcript level was calculated using the 2^-ΔΔCt method. The primer sequences used to amplify each transcript are listed in S1 Table.

### Western blotting and ELISA

hBMVEC were grown to confluency in 35mm dishes, then treated with 20 μM PBT434 or PBT434-met for 24h at 37°C, after which they were washed twice with ice-cold PBS containing 1mM CaCl_2_ and 0.5mM MgCl_2_ (PBS/Ca^2+^/Mg^2+^) and lysed by scraping in NP-40 lysis buffer (25 mM Tris, 150 mM NaCl, 1% Nonidet P-40, 0.1% SDS, pH 7.4) supplemented with 4X Halt™ protease inhibitor cocktail (Thermo Scientific). The cell suspension was incubated on ice for 15min, then centrifuged at 13,000 x g for 10 min at 4°C and the supernatant was collected as the cell lysate. Protein content was quantified by BCA assay (Thermo Scientific) and equal amounts of protein (20 μg) were mixed with 6X SDS-loading buffer to a final concentration of 1X and denatured at 50°C for 30min for SDS-PAGE. Samples were fractionated on a 4–15% SDS-polyacrylamide gradient gel, followed by transfer to a PVDF membrane using the mixed MW program on the Trans-Blot Turbo transfer system (Bio-Rad). Membranes were blocked in TBST (20 mM Tris, 150 mM NaCl, 0.05% Tween 20) containing 5% BSA (Gemini Bio, Sacramento, CA) at room temperature for 1h, followed by incubation with the primary antibody overnight at 4°C. Primary antibodies were diluted in 1% BSA in TBST as follows: 1:1000 rabbit anti-Fpn antibody (Cat # NBP1-21502; Novus Biologicals, Centennial CO), 1:800 goat anti-TfR (polyclonal, Cat # AF2474, R&D Systems), 1:1000 goat anti-Cp (polyclonal, Cat # A80-124A, Bethyl Laboratories), 1:1000 rabbit anti-βactin (Cat # 4790, Cell Signaling Technologies). After washing, membranes were incubated at room temperature for 1h with 1:5000 secondary goat anti-rabbit HRP (NBP2-30348H, Novus) in 3% BSA in TBST. Blots were visualized using SuperSignal West Dura extended duration substrate (Thermo Scientific) on a ChemiDoc™ Touch Imager (Bio-Rad).

For ferritin ELISA assay, hBMVEC were loaded with 1 μM Fe^2+^-citrate using a reductase-independent uptake protocol with the addition of 250 μM citrate and 5mM ascorbate in RPMI+serum growth media. After 24h loading, cells were washed and treated in the absence or presence of PBT434 for an additional 1hr, then quenched with ice-cold quench buffer [50] and lysed in NP-40 lysis buffer at 37°C for 1h. The ELISA assay was performed according to manufacturer’s instructions (Cat # FRR31-K01, Eagle Biosciences, Amherst NH), in duplicate or triplicate. Results were analyzed and normalized to protein content determined by BCA assay as described above.

For transferrin ELISA, hBMVEC were grown in 35mm tissue culture dishes until confluency, then treated with 20 μM PBT434 for 24h in RPMI-serum. Cells were lysed and assayed for protein content as described above, and lysates were assayed for transferrin receptor by ELISA assay using the Human TfR/Transferrin R/CD71 ELISA Kit (Cat # EH448RB, Thermo), in duplicate. Results were normalized to total protein content.

For ceruloplasmin ELISA, hBMVEC or HepG2 cells were grown in 6cm tissue culture dishes for 24h in RPMI+serum, then switched to RPMI-serum in the absence or presence of PBT434 for an additional 24h. Media was removed, and cells were treated with 0.5 U/ml PI-PLC in PBS for 1h to release cell surface GPI-anchored proteins. Following incubation, PI-PLC-treated conditioned media was collected, spun down to remove cells, 0.2 μm filter sterilized, and then concentrated using a 10K MWCO Amicon centrifugal filter. The ELISA assay was performed using the human ceruloplasmin ELISA kit (Cat # NBP2-60616, Novus) according to manufacturer’s instructions, in duplicate. Results were normalized to protein content as described above.

### ^55^Fe^2+^ accumulation and efflux assays

For accumulation assays, hBMVEC monolayers were loaded with 1 μM ^55^Fe^2+^-citrate using a reductase-independent uptake protocol with the addition of 250 μM citrate and 5mM ascorbate in RPMI+serum growth media. Reactions were quenched with ice-cold quench buffer as previously described [50], and lysed in lysis buffer at 37°C for 1h. Lysates were assayed for cell-associated ^55^Fe counts (Beckman LS6500 Scintillation Counter) and normalized to protein content determined by BCA assay (Thermo Scientific, Waltham, MA). ^55^Fe-transferrin was prepared as previously described [50]. The resulting ^55^Fe-Tf solution was buffer exchanged using an Amicon Ultra 10K MWCO Centrifugal Filter (EMD Millipore, Burlington, MA) to remove any remaining non-Tf bound ^55^Fe. This procedure yielded a ^55^Fe-Tf purity of >95%.

To determine Fpn-dependent iron efflux, cells were loaded with 1 μM ^55^Fe^2+^-citrate in RPMI+serum in the presence or absence of 1.4 μM mini-hepcidin PR73 for 24h at 37°C. Following ^55^Fe^2+^ loading, cells were washed twice with pre-warmed RPMI media containing 250 μM citrate, and efflux was initiated in the continued presence or absence of PR73 and the presence or absence of 20 μM PBT434 included in the efflux media for 5h. After 5h, the cells were quenched, lysed and processed for counting as described above.

### FITC-dextran permeability

Barrier integrity was also monitored by permeability to FITC-dextran (4kD, Sigma). hBMVEC were plated in transwell inserts in RPMI+serum media for 2 days. On day 2 post-plating, cells were polarized with RPMI+serum in the apical chamber and RPMI-serum in the basal chamber. On day 3, cells were loaded with 250 μg/ml FITC-dextran in the apical chamber in RPMI+serum and RPMI-serum in the basal chamber. At the indicated timepoints, 50μl basal media, in triplicate, was transferred to a black-sided 96-well plate and fluorescence intensity (exc. 485, em. 520nm) was measured using a FLUOStar Omega plate reader. Following the assay, cells were lysed and assayed for protein content as described above. A FITC-dextran standard curve was used to determine the amount of FITC-dextran (μg) that permeated into the basal chamber, and this value was normalized to the protein content (mg). TEER was monitored starting 1 day post-plating until the start of the assay and continued for the duration of the assay, with measurements taken at each indicated timepoint (**S1 Fig**).

### Statistical analysis

All statistical analyses were performed using Prism 8 (GraphPad Prism software, San Diego, CA). Data are represented as mean ± SEM, and n is equivalent to the number of biological replicates for each condition unless otherwise noted. Comparisons between two conditions (one variable) were made using unpaired *t* test. Comparisons between multiple samples were made using one-way ANOVA statistical analyses. For comparison of samples between 2 groups (two variables), two-way ANOVA statistical analysis was used.

## Supporting information

S1 Fig Barrier integrity of hBMVEC is unaffected by PBT434 treatment. (**A**) hBMVEC in transwells were monitored for barrier integrity by TEER over 4 days post-plating, reported as Ω∙cm^2^. Data are represented as mean ± SEM, n = 6 or 12 biological replicates. Statistical significance was determined using one-way ANOVA and Tukey’s multiple comparison test. *, statistically significant compared to day 1 or as indicated; ns, not statistically significant; *, p < 0.05; ****, p < 0.0001. (**B**) hBMVEC barrier integrity was monitored in the presence or absence of PBT434 (20 μM) starting after barrier formation on day 3 post-plating. TEER was measured at the indicated timepoints and represented as Ω∙cm^2^. Data are represented as mean ± SEM, n = 6 or 12 biological replicates. Statistical significance was determined using unpaired t-test, ns, not statistically significant. (**C**) hBMVEC barrier integrity was monitored by FITC-dextran impermeability in the presence or absence of PBT434 (20 μM) starting on day 3 post-plating. Cells were loaded with 250 μg/ml FITC-dextran in the apical chamber for 24h, and the basal chamber media was sampled at the indicated timepoints. The amount of FITC-dextran that permeated the barrier (μg FITC-dextran/mg protein) was compared between control and PBT434-treated cells. TEER was monitored for the duration of the assay, shown in (B). Data are represented as mean ± SEM, n = 6 biological replicates/condition. Statistical significance was determined using unpaired t-test, ns, not statistically significant.
(TIF)

S2 Fig Serum suppresses the apparent difference in apical vs basal uptake rates of ^14^C-PBT434. hBMVEC in transwells were assayed for ^14^C-PBT434 apical vs basal trajectory as described in Fig 4, but with RPMI *with serum* in both the apical and basal chambers. Rates of ^14^C-PBT434 uptake or efflux were calculated using linear regression analysis. Statistical significance was determined by Welch’s t-test, ns, not statistically significant.
(TIF)

S3 Fig GPI-Cp is detected in HepG2 but not hBMVEC. hBMVEC or HepG2 were grown in monolayers for 24h media with serum, then incubated in the absence or presence of PBT434 (20 μM) in media without serum for an additional 24h. Following incubation, cells were treated with PI-PLC (0.5 U/ml) for 1hr to release any GPI-anchored cell surface proteins. Media was collected, concentrated, assayed for Cp protein by sandwich ELISA and normalized to total protein content. Data are represented as mean ± SEM, n = 4 biological replicates. Statistical significance was tested using t-test; ns, not statistically significant; ****, p < 0.0001, compared to control. The lower sensitivity limit of this kit is reported to be 0.12 ng/ml (or 0.0088 ng/mg protein average) indicated by the dashed line.
(TIF)

S4 Fig Pre-treatment with PBT434, but not PBT434-met, elevated accumulated ^55^Fe^2+^ uptake for at least 24h. hBMVEC were pre-treated with 20 μM PBT434 or PBT434-met for 24h prior to loading with 1 μM ^55^Fe^2+^ (250 μM citrate, 5mM ascorbate) for up to 24h. The amount of ^55^Fe^2+^ accumulated in lysates was assayed at 6h (**A**) and 24 h (**B**). Data are presented as mean ± SEM, n = 4 biological replicates. Statistical significance was determined using one-way ANOVA and Tukey’s multiple comparison test; **, p < 0.01; ***, p < 0.001 compared as indicated. The rates of ^55^Fe^2+^ accumulation between 6 and 24h were determined using linear regression analysis (**C**), and there was no statistically significant difference as determined by one-way ANOVA and Tukey’s multiple comparison test.
(TIF)

S1 Table Primers used for RT-qPCR. (DOCX)

S1 Raw images (PDF)
