# Supplementary figures and images for: The iron chelator, PBT434, modulates transcellular iron trafficking in brain microvascular endothelial cells
Source: PLoS One. 2021 Jul 26;16(7):e0254794. doi: 10.1371/journal.pone.0254794 (PMC8312958; doi:10.1371/journal.pone.0254794)

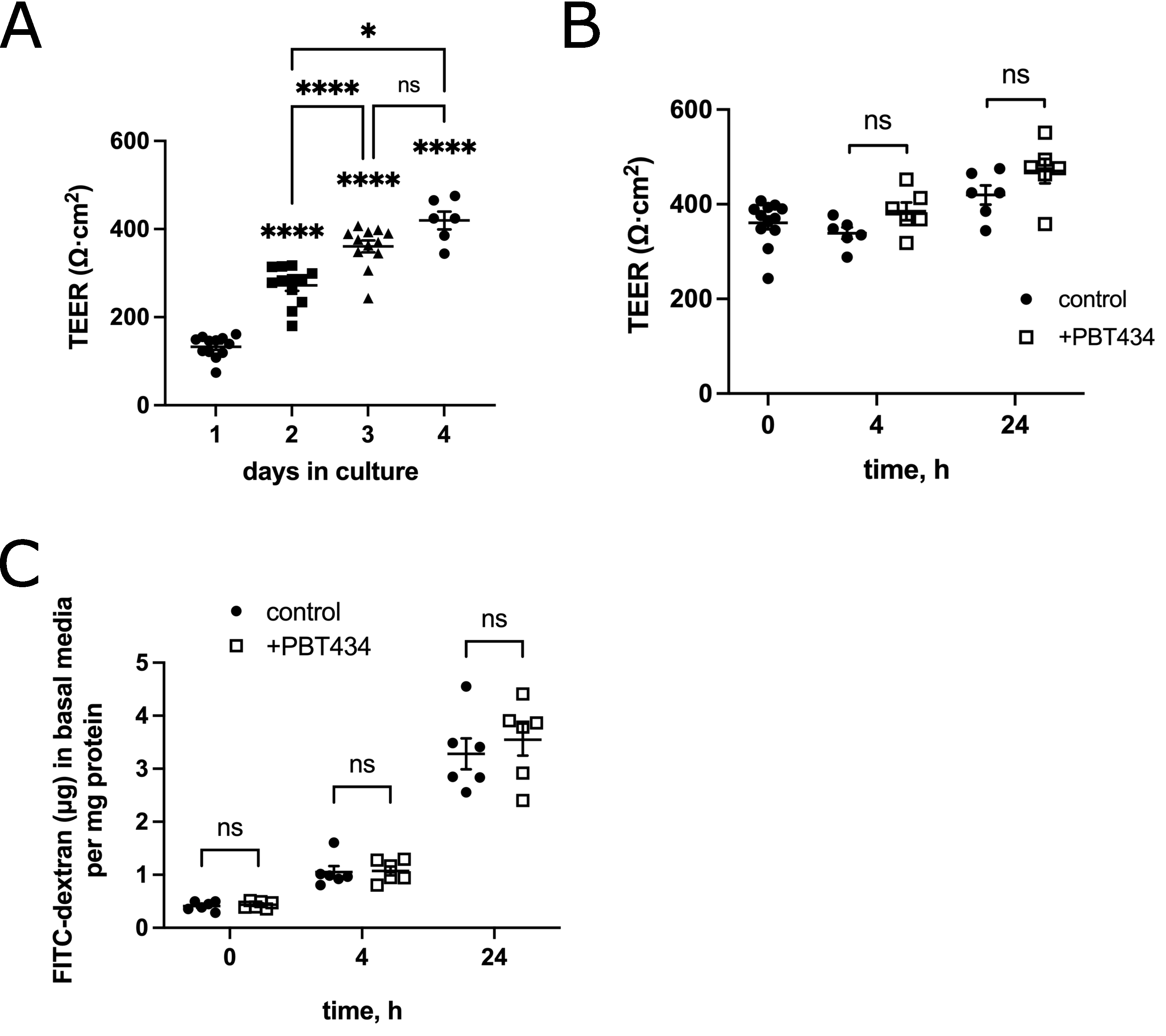

Supplement: S1 Fig — (A) hBMVEC in transwells were monitored for barrier integrity by TEER over 4 days post-plating, reported as Ω∙cm2. Data are represented as mean ± SEM, n = 6 or 12 biological replicates. Statistical significance was determined using one-way ANOVA and Tukey’s multiple comparison test. *, statistically significant compared to day 1 or as indicated; ns, not statistically significant; *, p < 0.05; ****, p < 0.0001. (B) hBMVEC barrier integrity was monitored in the presence or absence of PBT434 (20 μM) starting after barrier formation on day 3 post-plating. TEER was measured at the indicated timepoints and represented as Ω∙cm2. Data are represented as mean ± SEM, n = 6 or 12 biological replicates. Statistical significance was determined using unpaired t-test, ns, not statistically significant. (C) hBMVEC barrier integrity was monitored by FITC-dextran impermeability in the presence or absence of PBT434 (20 μM) starting on day 3 post-plating. Cells were loaded with 250 μg/ml FITC-dextran in the apical chamber for 24h, and the basal chamber media was sampled at the indicated timepoints. The amount of FITC-dextran that permeated the barrier (μg FITC-dextran/mg protein) was compared between control and PBT434-treated cells. TEER was monitored for the duration of the assay, shown in (B). Data are represented as mean ± SEM, n = 6 biological replicates/condition. Statistical significance was determined using unpaired t-test, ns, not statistically significant. (TIF) [file pone.0254794.s001.tif]

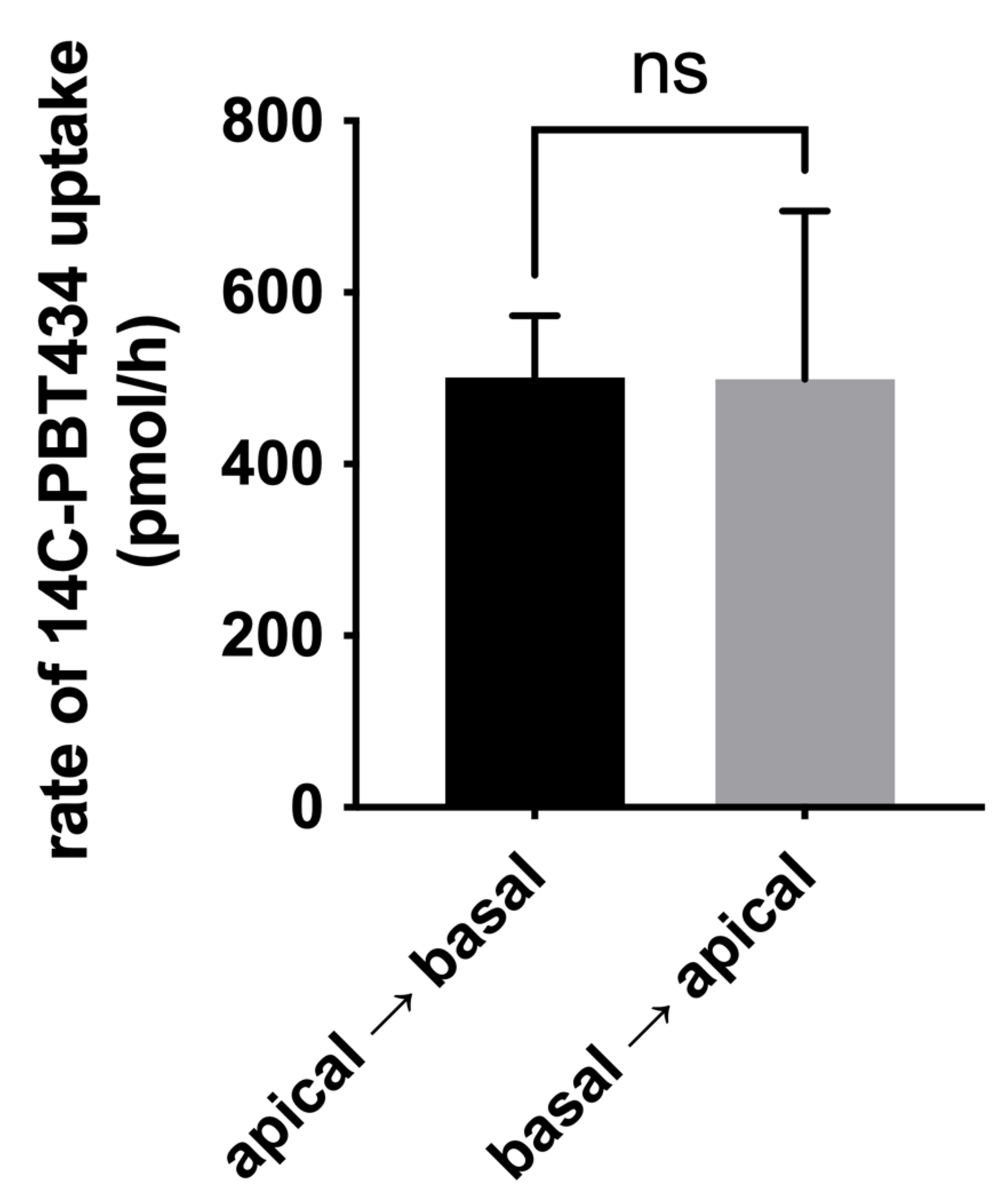

Supplement: S2 Fig — hBMVEC in transwells were assayed for 14C-PBT434 apical vs basal trajectory as described in Fig 4, but with RPMI with serum in both the apical and basal chambers. Rates of 14C-PBT434 uptake or efflux were calculated using linear regression analysis. Statistical significance was determined by Welch’s t-test, ns, not statistically significant. (TIF) [file pone.0254794.s002.tif]

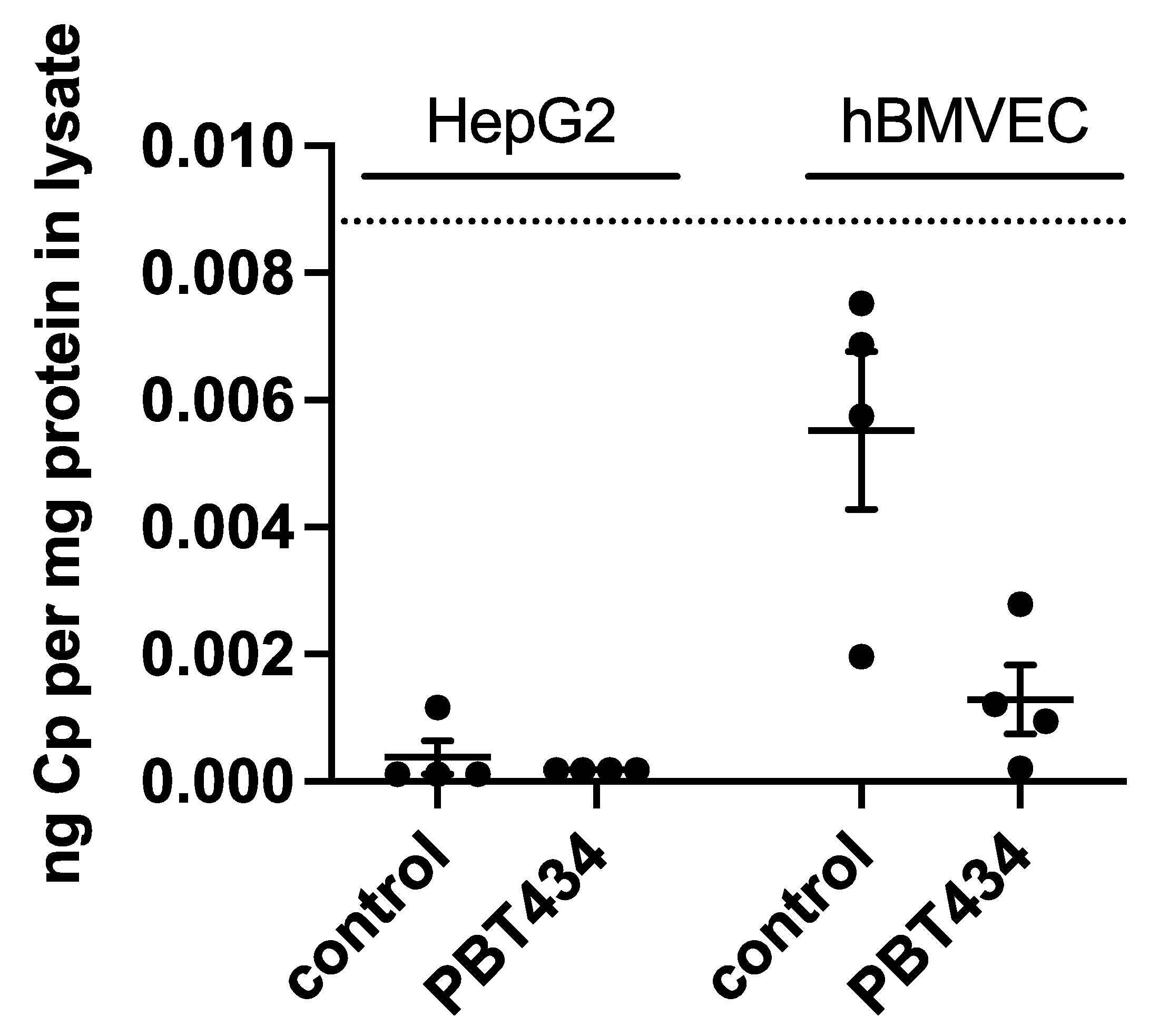

Supplement: S3 Fig — hBMVEC or HepG2 were grown in monolayers for 24h media with serum, then incubated in the absence or presence of PBT434 (20 μM) in media without serum for an additional 24h. Following incubation, cells were treated with PI-PLC (0.5 U/ml) for 1hr to release any GPI-anchored cell surface proteins. Media was collected, concentrated, assayed for Cp protein by sandwich ELISA and normalized to total protein content. Data are represented as mean ± SEM, n = 4 biological replicates. Statistical significance was tested using t-test; ns, not statistically significant; ****, p < 0.0001, compared to control. The lower sensitivity limit of this kit is reported to be 0.12 ng/ml (or 0.0088 ng/mg protein average) indicated by the dashed line. (TIF) [file pone.0254794.s003.tif]

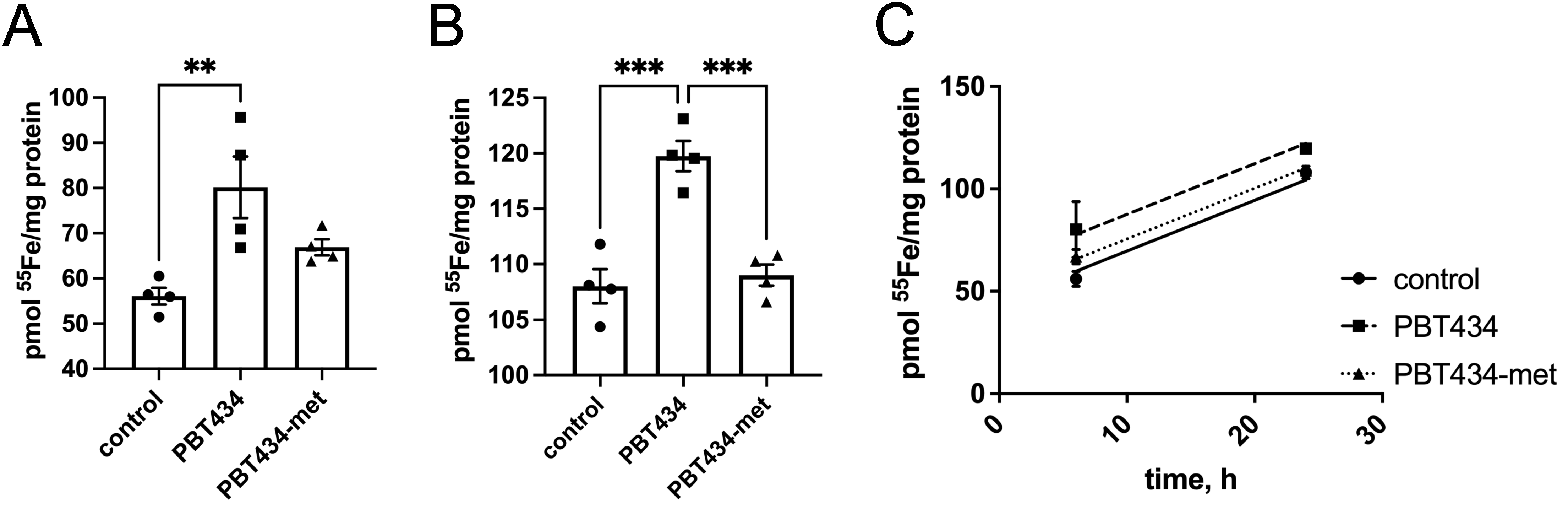

Supplement: S4 Fig — hBMVEC were pre-treated with 20 μM PBT434 or PBT434-met for 24h prior to loading with 1 μM 55Fe2+ (250 μM citrate, 5mM ascorbate) for up to 24h. The amount of 55Fe2+ accumulated in lysates was assayed at 6h (A) and 24 h (B). Data are presented as mean ± SEM, n = 4 biological replicates. Statistical significance was determined using one-way ANOVA and Tukey’s multiple comparison test; **, p < 0.01; ***, p < 0.001 compared as indicated. The rates of 55Fe2+ accumulation between 6 and 24h were determined using linear regression analysis (C), and there was no statistically significant difference as determined by one-way ANOVA and Tukey’s multiple comparison test. (TIF) [file pone.0254794.s004.tif]
