# Supplementary material for: The iron chelator, PBT434, modulates transcellular iron trafficking in brain microvascular endothelial cells
Source: PLoS One. 2021 Jul 26;16(7):e0254794. doi: 10.1371/journal.pone.0254794 (PMC8312958; doi:10.1371/journal.pone.0254794)
Supplement: S1 Table — (DOCX) [file pone.0254794.s005.docx]

**S1 Table. Primers used for RT-qPCR.**

| Transcript | Forward Primer | Reverse Primer |
| --- | --- | --- |
| β-actin | GGGTCAGAAGGATTCCTATG | GGTCTCAAACATGATCTGGG |
| Fpn | TCAGTTTGCAACATGTCTGTACC | GCAACGTATTGCAGTCTCCAT |
| Hp | TGGAGTGGTTTGGTACCAACATCG | TCTCCAGGCTCCAGATGTTACTGT |
| Non-isoform specific Cp | TGGTTTATCGTGAGTACACAG | TCTGCCCAAATGACAGGACC |
| PCBP1 | AATCAATGCCAGGCTTTCCTC | TTAAAACCTGGAATTACCGACCAG |
| PCBP2 | AATCAATGCCAGGCTTTCCTC | TTAAAACCTGGAATCGCTGACTG |
