# Supplementary material for: The iron chelator, PBT434, modulates transcellular iron trafficking in brain microvascular endothelial cells
Source: PLoS One. 2021 Jul 26;16(7):e0254794. doi: 10.1371/journal.pone.0254794 (PMC8312958; doi:10.1371/journal.pone.0254794)
Supplement: S1 Raw images — (PDF) [file pone.0254794.s006.pdf]

PONE-D-20-39399

Supplemental Materials - Original raw images for Western blots

1. Raw images for Figure 7A, top panel.

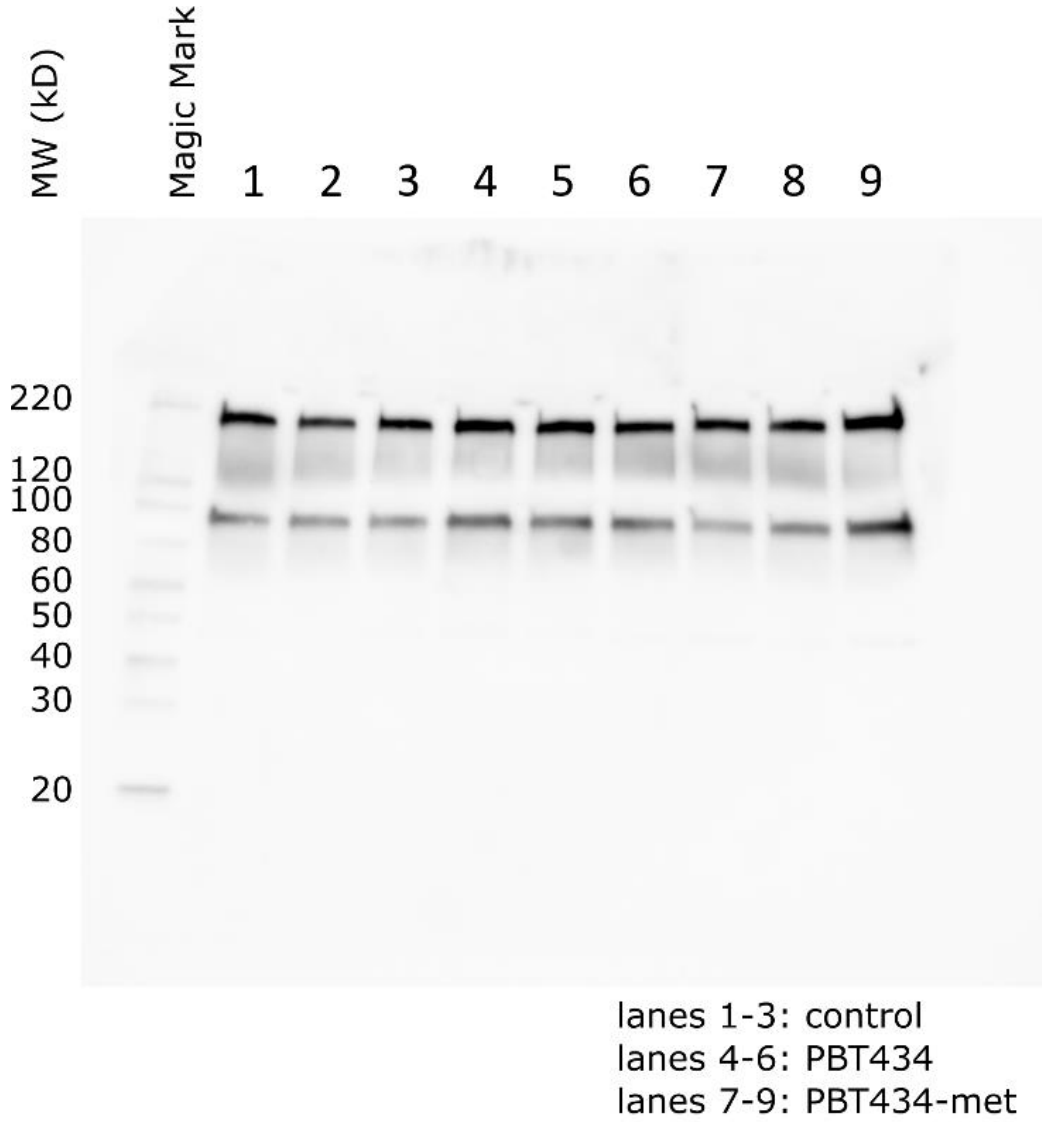

Western blot of transferrin receptor (α-TfR, R&D Systems; monomer: 95kD, homodimer: 180-200kD)

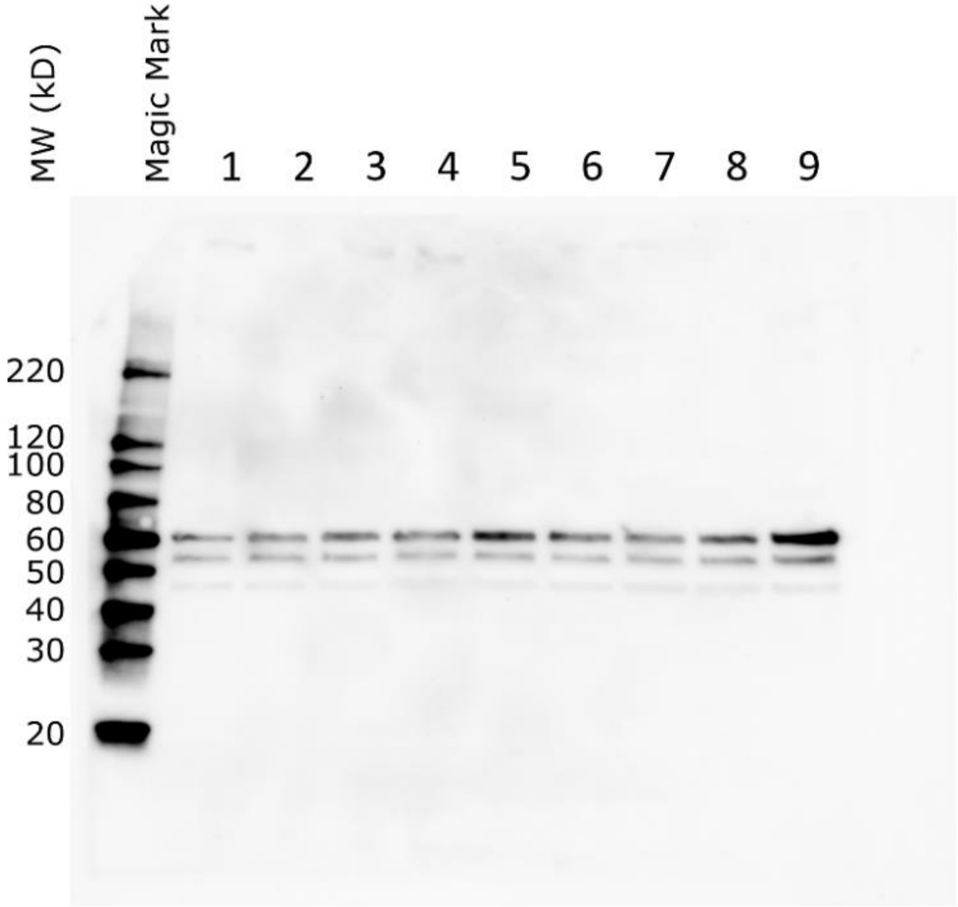

lanes 1-3: control
lanes 4-6: PBT434
lanes 7-9: PBT434-met

Western blot of ferroportin (α-Fpn, Novus, 55-60kD)

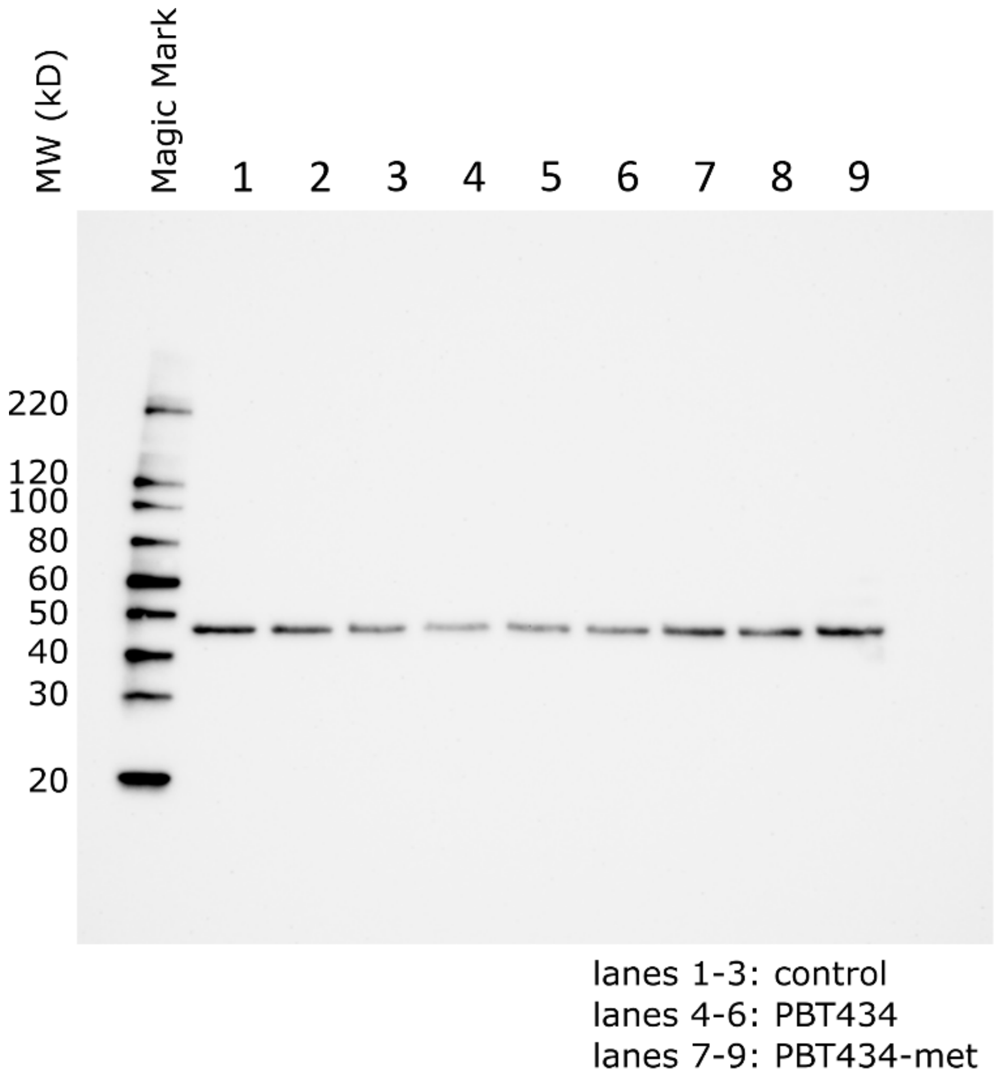

Western blot of beta-actin (α-βactin, Cell Signaling, 45kD)

2. Raw images for Figure 7A, bottom panel.

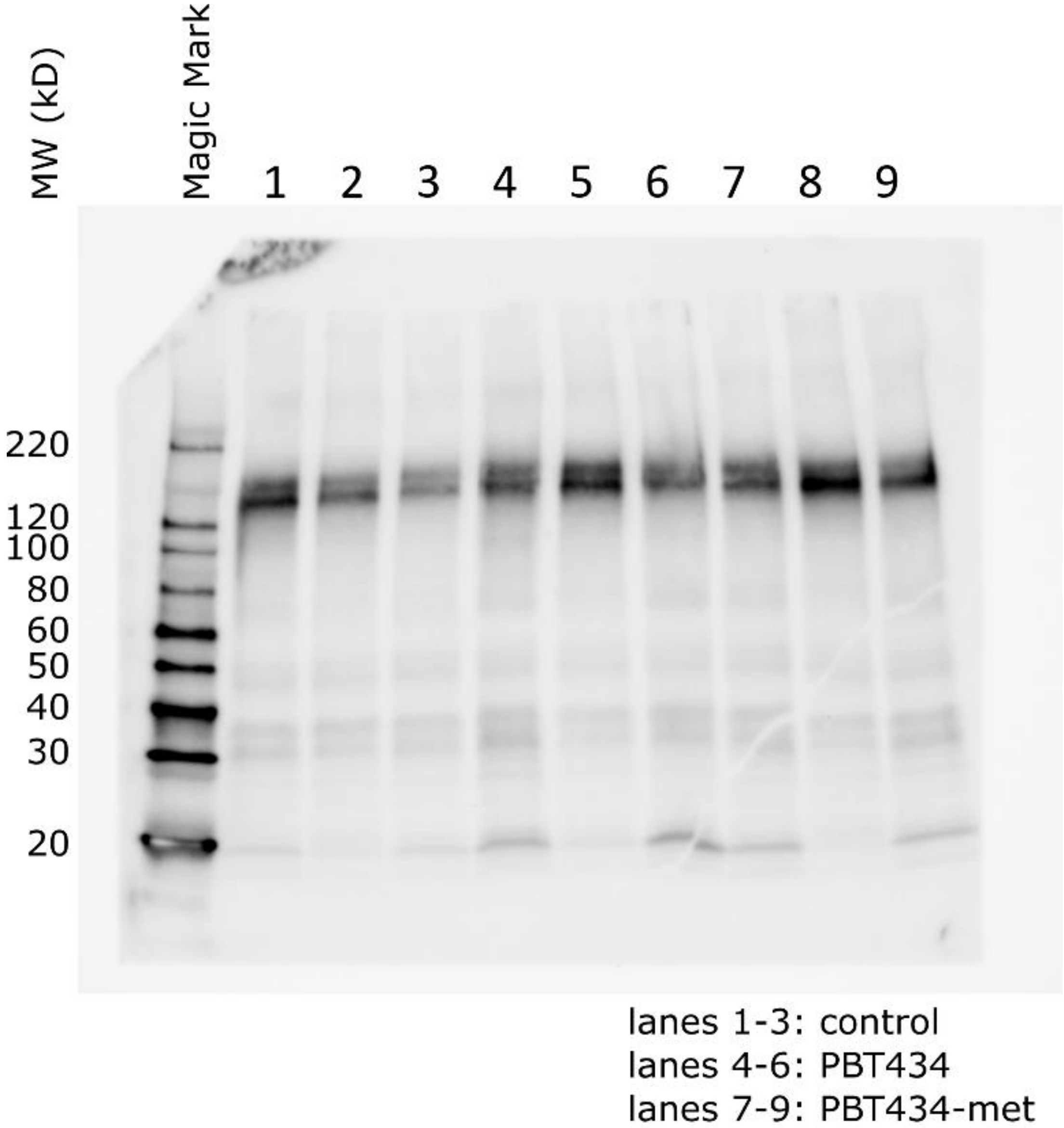

Western blot of cerulosplamin (α-Cp, Bethyl, 130-150 kD)

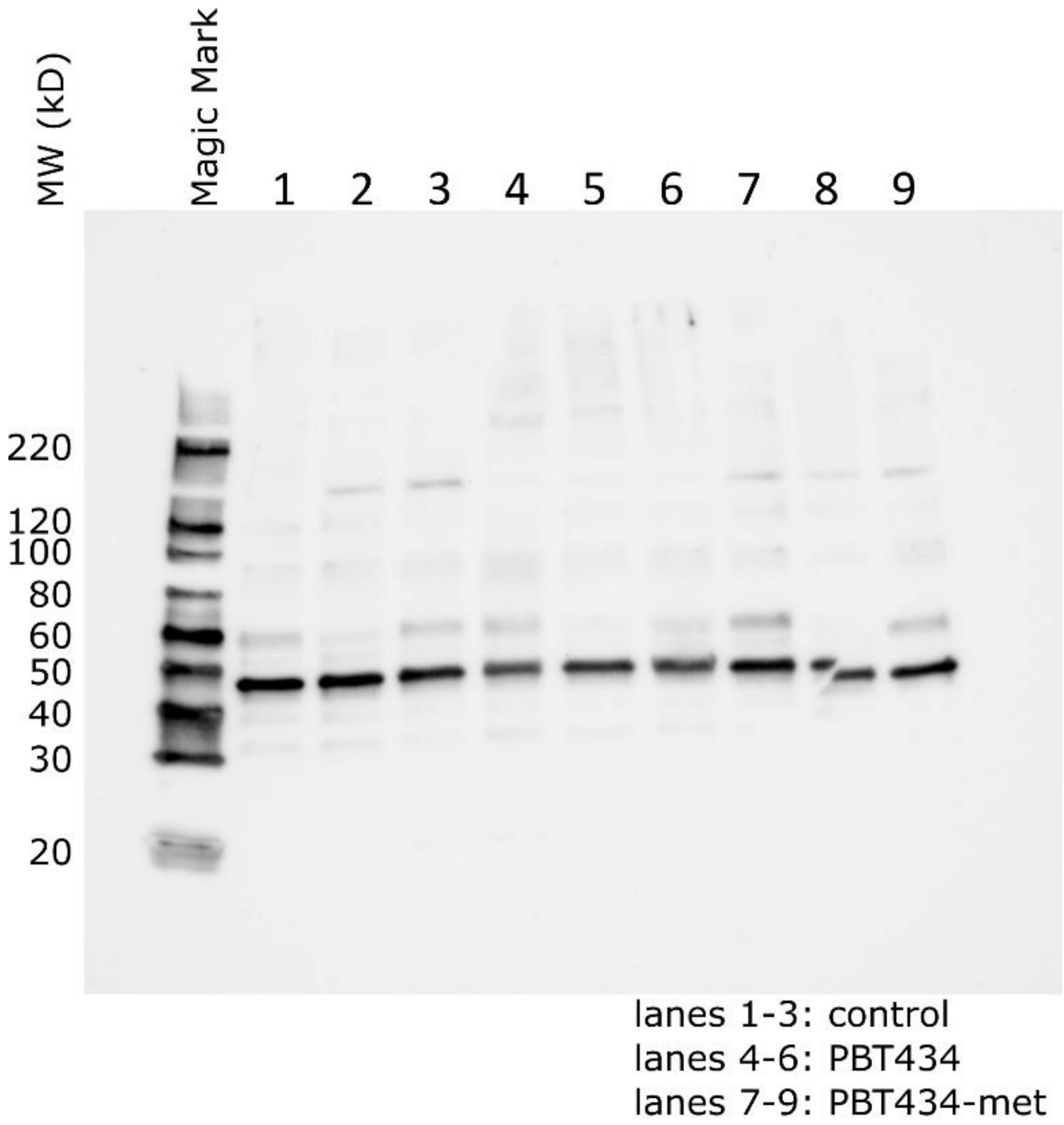

Western blot of beta-actin (α-Bactin, Cell Signaling, 45 kD)

3. Additional raw images for western blots used in quantification in Figure 7.

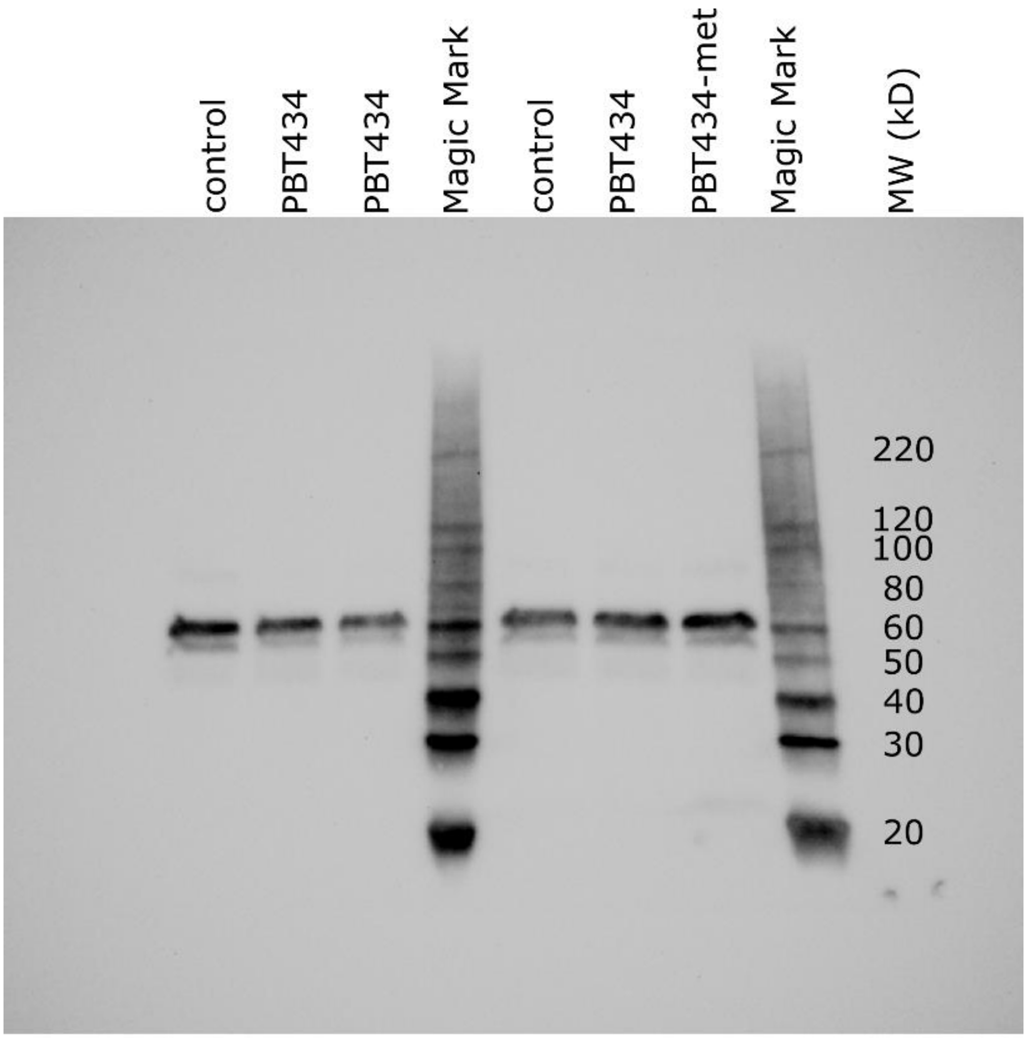

Western blot of ferroportin (α-Fpn, Novus, 55-60kD)

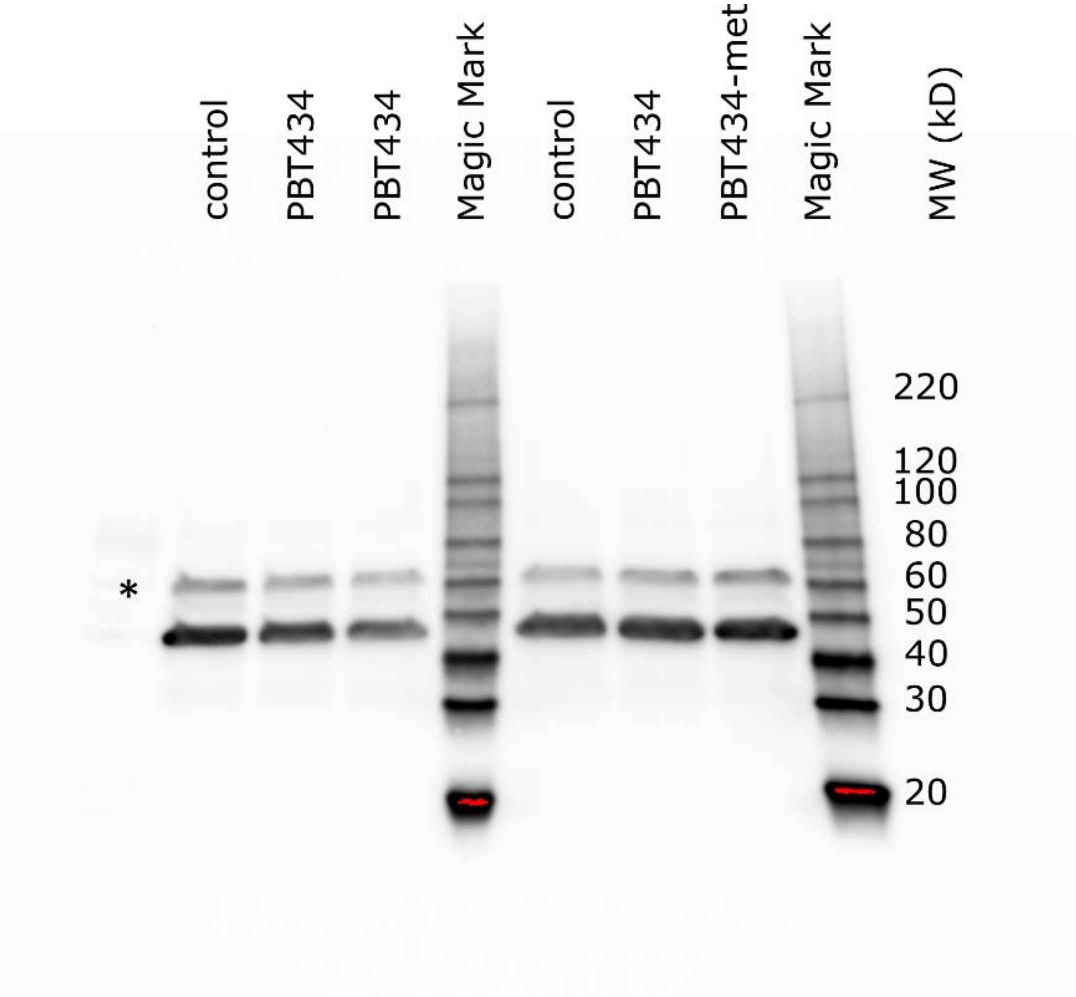

Western blot of beta-actin (α-βactin, Cell Signaling, 45kD)
* indicates non-specific band leftover from α-Fpn
